# The effect of non-pharmacological interventions on bone health among patients with low bone mass: a systematic review and meta-analysis

**DOI:** 10.3389/fendo.2025.1612739

**Published:** 2025-12-10

**Authors:** Xiaona Na, Yucheng Yang, Huanhuan Yang, Zekun Chen, Xiaochen Qu, Jian Zhang, Mo Chen, Dantong Wang, Denis Breuille, Kai Yu, Ai Zhao, Zhihui Li

**Affiliations:** 1 Vanke School of Public Health, Tsinghua University, Beijing, China; 2 Institute for Healthy China, Tsinghua University, Beijing, China; 3 Curtin School of Population Health, Curtin University, Perth, WA, Australia; 4 Nestlé Institute of Health Sciences China Hub, Nestlé Research, Beijing, China; 5 Nestlé Institute of Health Sciences, Nestlé Research, Lausanne, Switzerland

**Keywords:** exercise, diet, life style, low bone mass, systematic review, meta-analysis

## Abstract

**Background and objective:**

Low bone mass represents a critical period for “watchful waiting” interventions to prevent osteoporosis. This systematic review provides a comprehensive overview of non-pharmacological interventions for patients with low bone mass.

**Methods:**

We included randomized controlled trials (RCTs) investigating the efficacy of non-pharmacological interventions for improving bone health outcomes in participants with low bone mass. Publications were collected from three databases. A meta-analysis was performed for outcomes reported in three or more articles, with changes in outcomes expressed as mean differences (MD) or standardized mean differences (SMD) with 95% confidence intervals (CIs).

**Results:**

A total of 26 eligible articles were included. Exercise interventions increased serum osteocalcin levels (SMD = 1.26, 95% CI: 0.22–2.31) compared to the control group. Narrative synthesis of studies showed a protective effect of exercise on lumbar spine and femoral neck BMD. For nutrition interventions, polyphenol extracts showed efficacy in improving lumbar spine BMD. The results of collagen supplements were inconsistent, and the effects of micronutrients were limited.

**Conclusion:**

In conclusion, more evidence from RCTs, particularly those investigating comprehensive lifestyle interventions and tailored prevention for moderate and severe low bone mass, especially among older men, is necessary.

## 1 Introduction

The World Health Organization defined osteoporosis in 1994 as a “progressive systemic skeletal disease characterized by low bone mass and microarchitectural deterioration of bone tissue, with a consequent increase in bone fragility and susceptibility to fracture” (1). Recently, multimodal and comprehensive approaches have been recommended for diagnosing osteoporosis, incorporating individual fracture risk, clinical history, physical examination, suggestive symptoms (e.g., height loss, back pain, and/or fractures), and vertebral imaging (2, 3). The prevalence of osteoporosis worldwide is estimated to be 18.3%, with higher rates among women (23.1%) compared to men (11.7%) (4). It is a leading cause of fractures and is associated with increased mortality risk among older individuals, making it a significant global public health concern (5–7).

Low bone mass—often referred to as osteopenia in postmenopausal women and men aged ≥ 50 years—is an intermediate stage between normal bone mineral density (BMD) and osteoporosis (3). It is characterized by a T-score derived from BMD value that falls more than 1 standard deviation (SD) but less than 2.5 SD below the BMD of the reference population. As the condition progresses, it reaches the diagnostic threshold for osteoporosis, defined by a T-score of BMD less than -2.5 (8). The prevalence of low bone mass was estimated to be 40.4% in the population aged over 50 years, more than double the prevalence of osteoporosis (9). Moreover, in women, it is estimated that more than 50% of fragility factures occur in those with low bone mass rather than osteoporosis (10). Thus, low bone mass as the pre-clinical stage of osteoporosis serves as a critical window for interventions aimed at slowing down the progression of low bone mass and reducing the risk of osteoporosis and fractures (11).

Pharmaceutical agents that target BMD are commonly used as a first-line treatment of osteoporosis, with their effect in reducing fracture risk by 30% to 50% (12). However, these medications are typically limited to osteoporotic patients, despite studies also showing that pharmacological treatment with zoledronate significantly reduced the risk of fractures in older women with low bone mass (2). In addition, barriers to adoption of pharmaceutical treatment to prevent and treat osteoporosis have been identified from both the perspective of patients and physicians (13). These factors further contribute to the risk of fracture in individuals with low bone mass.

Non-pharmacological management of osteoporosis mainly involves lifestyle modifications, including adopting healthier diets, calcium and vitamin D supplementation, adequate weight-bearing exercise, smoking cessation, and avoidance of excessive alcohol consumption (2). From a practitioner perspective, the stage of low bone mass is considered a period of “watchful waiting” to prevent transition into osteoporosis (14). Although direct evidence comparing intervention responses between populations with low bone mass and others (e.g., those with normal BMD or established osteoporosis) is limited, some studies provide indirect evidence suggesting that the effects may differ between these populations. For example, a trial found that the effect of exercise training on BMD changes depended on baseline BMD among postmenopausal women without osteoporosis treatment (including those with low bone mass and normal BMD), indicating lifestyle interventions may have greater effects among individuals with low BMD (15). Another systematic review and meta-analysis of randomized controlled trials (RCTs) showed that the effect of exercise on BMD varied across different health status and body sites (16). On the other hand, unlike in osteoporosis, where medications are essential for fracture prevention and non-pharmacological strategies serve as supplements, this may not be the case for low bone mass (3, 17, 18). Compared with individuals with established osteoporosis, people with low bone mass may retain greater residual bone remodeling capacity and exhibit higher adherence to lifestyle interventions due to the absence of overt disease or medication burden (19). To develop evidence-based approaches for maintaining bone health, preventing osteoporosis, and reducing the burden of fractures, it is important to understand the potential benefits and limitations of non-pharmacological treatments in addressing low bone mass. However, to date, there has been no comprehensive systematic review that specifically examines the effect of lifestyle interventions on low bone mass, despite existing reviews reporting benefits of exercise or nutritional interventions on bone health in mixed populations without distinguishing between osteopenia and osteoporosis (20, 21).

To fill this knowledge gap, the objective of this systematic review was to synthesize evidence from RCTs on the effects of non-pharmacological lifestyle interventions in populations with low bone mass. By focusing specifically on this population, we aimed to provide a comprehensive evaluation of intervention efficacy and limitations, thereby offering valuable insights for clinicians, researchers, and policymakers.

## 2 Materials and methods

We conducted a systematic review and meta-analysis following the guidelines outlined in the Preferred Reporting Items for Systematic Review and Meta-Analyses (PRISMA) statement (**Supplementary Table S1**) (22). This study has been registered in PROSPERO (CRD42023466865).

### 2.1 Data sources and searches

The search strategy can be found in **Supplementary Table S2**. To ensure comprehensive coverage of relevant literature, we adopted a deliberately broad search approach. Searches were conducted for articles in PubMed, Web of Science, Embase, and CINAHL, spanning from the inception of the databases to May 17th, 2023, updated on January 28th, 2024. The search strategy employed a combination of free-text and Medical Subject Headings (MeSH) terms, involving a comprehensive set of terms related to the low bone mass (low bone mass, low BMD, osteopenia, etc.), “non-pharmacological treatment”, “lifestyle”, specific lifestyle factors (exercise, physical activity, sport, nutrition, dietary, food, supplement, smoking, alcohol, etc.), “randomized controlled trial (RCT)”, and “adults”. EndNote X9 was used for reference storage and management. Each retrieved record was independently assessed for eligibility by two reviewers, with any discrepancies resolved by consultation with a third reviewer.

### 2.2 Study selection

Specific inclusion criteria are detailed in **Table 1**. We included RCTs investigating the efficacy of non-pharmacological interventions in enhancing bone health outcomes in participants with low bone mass published in English. In cases where multiple publications reported overlapping data from the same RCT, the study with the largest sample size or the longest intervention duration was selected to avoid duplicate inclusion of participants.

**Table 1:** PICOS criteria in this study.

Item	Inclusions	Exclusions
Participants	Participants aged ≥ 18 years with low bone mass	Participants with osteoporosis, fracture, normal BMD, or secondary low bone mass, or who were pregnant or lactating
Interventions	Any non-pharmacological interventions, such as dietary modifications, exercise, smoking and alcohol cessation, health education programs, and any other relevant interventions	Interventions involving pharmacological treatment
Comparisons	Control group with placebo or no intervention/treatment	Positive control (participants in control group received a known effective treatment)
Outcome	Any bone health outcomes, such as BMD and BTM	Outcomes not related to bone health
Study design	RCT in design	Any other types of studies

BMD, body mineral density; BTM, bone turnover marker; RCT, randomized controlled trial.

### 2.3 Data extraction and quality assessment

Information from eligible articles was independently and directly extracted using standardized data extraction templates. The extracted data included the following information:

1. Study details: First author, title, and publication year;
2. Characteristics of the study and population: Sample size, study design, country where the study was conducted, baseline age of participants, and body sites of low bone mass;
3. Details of the lifestyle intervention: Type, content, dosage, frequency, and duration;
4. Measurement methods and value for outcomes, including baseline and post-intervention values, and change values, which available.

The revised Cochrane risk-of-bias tool for randomized trials (ROB 2) was used to assess the risk of bias of studies (23). This checklist consists of five domains: the randomization process, deviations from intended interventions, missing outcome data, measurement of the outcome, and selection of the reported results. The overall bias of each study was determined as “low risk of bias”, “some concerns”, and “high risk of bias”.

### 2.4 Categorization of evidence levels and statistical analysis

An evaluation system was utilized to assess the effects of intervention and categorize evidence levels adapted from a previous systematic review (24). The results for bone health outcomes from each study were recorded, excluding the intervention that solely reported by one study. Briefly, intervention effects were classified as “positive (+)”, “negative (-)”, “no effect (0)”, and “inconsistent (?)” according to the percentage of RCTs reporting significant effects. A “positive”, “negative”, or “no effect” classification was assigned if ≥ 60% of studies reported the respective outcome, a threshold chosen *a priori* based on previous systematic reviews (24). Conversely, an “inconsistent” classification was assigned when none of either three categories reached 60%, or only one study reported the outcome. The “inconsistent” label was applied when results were mixed, or the number of studies was insufficient, to reduce the risk of drawing misleading conclusions.

A meta-analysis was conducted to synthesize data across multiple studies for outcomes reported in three or more articles. The mean difference (MD) with 95% CIs of change value from baseline to endpoint was used as the summary statistic for continuous data. If the MD and the standard deviation (SD) for change was not reported, they were calculated using **Equation 1** and **Equation 2**, as recommended by the Cochrane Handbook (25). This calculation assumed a correlation coefficient (R) of 0.5 for **Equation 2**. In cases where different assessment methods were employed or different unites that could not be unified across various articles, the standardized mean difference (SMD) was used as the summary statistic.

(1) $${\text{MD}}_{\text{change}}={\text{Mean}}_{\text{endpoint}}-{\text{Mean}}_{\text{baseline}}$$

(2) $${\text{SD}}_{\text{change}}=\sqrt{{\text{SD}}_{\text{ baseline}}^{2}+{\text{SD}}_{\text{ endpoint}}^{2}-(2\times \text{R}\times {\text{SD}}_{\text{ baseline}}\times {\text{SD}}_{\text{ endpoint}})}$$

If multiple time points were reported for the outcomes, data from the last time point were selected. Heterogeneity among studies was assessed using the *I*-square statistic (*I*^2^) and categorized as low (*I*^2^ ≤ 40%), moderate (40% < *I*^2^ ≤ 70%), and high (*I*^2^ > 70%) (26). Studies with *p*-values of ≥ 0.1 and *I*^2^ of ≤ 40% were estimated using a fixed-effects model; otherwise, the random-effects model was applied. Egger’s test was employed to explore potential publication bias. All meta-analyses were performed using *meta* package (version 7.0-0) in R 4.1.1 (R Core Team, Vienna, Austria). A *p*-value of <0.05 was considered statistically significant.

## 3 Results

### 3.1 Characteristics of included articles

**Figure 1:**
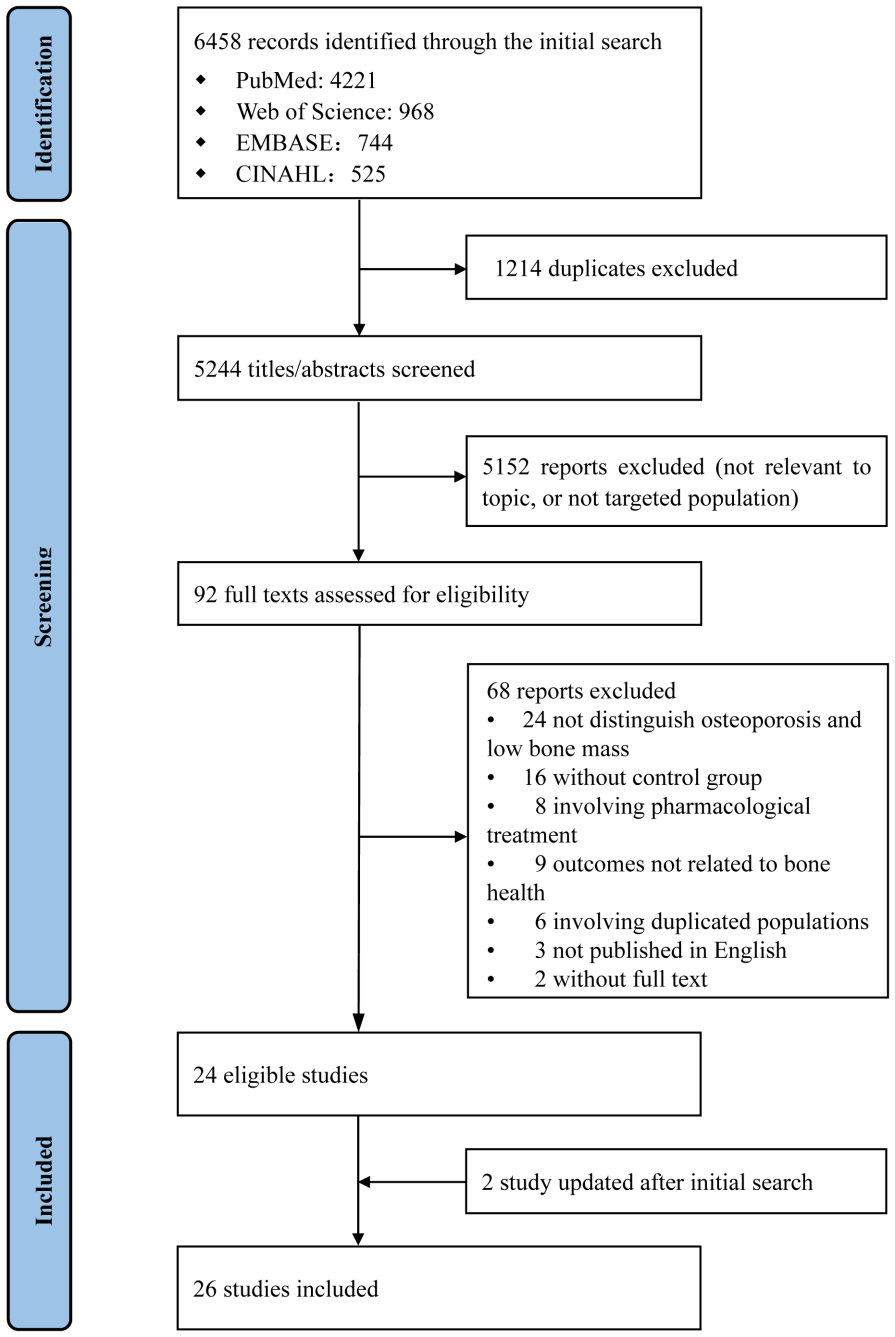
Flowchart of study identification and screening.

The study flowchart is shown in **Figure 1**. Twenty-six RCTs met the inclusion criteria: exercise alone (nine studies), nutrition alone (eighteen studies), and a comprehensive intervention combining exercise and nutrition (one study) (27–50). Among these articles, one study included multiple arms and investigated all three types of interventions. Most studies enrolled postmenopausal women; only one included both men and women. Overall, 2,143 individuals were included from 14 countries, across Europe (nine studies), Asia (six studies), North America (five studies), Oceania (two studies), and South America (two studies). Among them, 285 were allocated to exercise interventions, 853 to nutrition interventions, 37 to comprehensive interventions, and 968 to control groups.

The included studies assessed various bone health outcomes. BMD (g/cm²) at different skeletal sites was measured using dual-energy X-ray absorptiometry (DXA) (3). Two studies evaluated bone mineral content (BMC) at specific skeletal sites (51). Additionally, seventeen studies analyzed bone turnover markers (BTMs), including formation markers bone-specific alkaline phosphatase [BALP], osteocalcin [OC], and procollagen type I N-terminal propeptide [P1NP]), resorption markers (C-terminal telopeptide of collagen [CTX] and N-telopeptides of type I collagen [NTX], calcium and phosphorus metabolism markers (parathyroid hormone [PTH] and 25-hydroxyvitamin D [25-OH-D3]), as well as selected hormones and cytokines (interleukin 1 [IL-1], interleukin 6 [IL-6], tumor necrosis factor [TNF-α]). Detailed study characteristics are presented in **Table 2**.

**Table 2:** Basic characteristics of included studies.

Study	Year of implemented	Register number	Study design	Population characteristics	Age at baseline	Country	Region of interest at baseline	Measurement of bone	Diagnosis criteria of low bone mass	Intervention	Intervention frequency and duration	Control	Treatment for all arms^a^	Outcomes
Argyrou 2020	January 2017 to November 2018	NCT03999775	RCT	Postmenopausal women	Intervention group: 62.1 ± 6.3, placebo group: 62 ± 7.6 in	Greece	Lumbar spine or femur	DXA	BMD T-score between -1.5 and -2.5	N=21, a sachet containing 5 g bioactive collagen peptides daily	Daily for 3 months	N=22, placebo	500 mg of elemental calcium and 400 IU vitamin D3 daily	Serum P1NP and CTX
Basat 2013	November 2006 and May 2008	NA	RCT	Postmenopausal women	40–70	Turkey	Lumbar spine or FN	DXA	BMD T-score between -1.5 and -2.5	(1) N = 11, a 15-minute warm up, a 45 to 60-minute isometric strengthening, and a 10-minute cool down; (2) N = 12, a 15-minute warm up, a 45 to 60-minute high-impact exercises, and a 10-minute cool down.	One-hour exercise session, three times a week for 6 months	N=12, no training	Calcium (1200 mg) and vitamin D (800 IU)	L1–L4 spine and FN BMD, serum OC and NTX
Bolton 2012	March 2002 to August 2006	NA	Single-blind RCT	Postmenopausal women	over 50	Australia	Total hip	DXA	BMD T-score between -1.5 and -2.5	N=19, a 10-min warm-up and 10-min cool-down period, followed by 40 min of resistance training, impact loading and balance exercises.	Three times per week for 52 weeks with two one-week scheduled breaks	N=18, continue their usual care and levels of physical activity	Two tablets daily (300 mg of elemental calcium per tablet); participants with 25-OH-D levels below 60 nmol/L were advised to take a vitamin D supplement.	Total hip and total lumbar spine BMD, whole body and lower limb BMC, muscle strength, static and dynamic balance
Castelo-Branco 2015	NA	NA	Open-label RCT	Perimenopausal women	Under 55	Spain	Back and knee	DXA	BMD T-score between -1.5 and -2.0	N=33, daily 150 mg no-collagen proteins, 432 mg collagen, 356 mg calcium, and 164 mg phosphorus	Daily for 6 months	N=33, 1200 ml calcium	None	Basal pain and induced spine and knee pain
Cheung 2008	January 2002 to September 2004	NCT00150969	RCT	Postmenopausal women	Intervention group: 58.9 (40.1–80.5), Placebo group: 59.2 (46.1–82.3)	Canada	Lumbar spine (L1–L4), total hip, or FN	DXA	BMD T-score between -1.5 and -2.0	N=208, 5mg/day of vitamin K_1_, pills with food	Daily for 2 years	N=217, placebo	1500 mg of calcium and 800 IU of vitamin D per day	Serum total OC, CTX
Cúneo 2010	July 2005 and January 2008	13/05022002	Double-blind RCT	Postmenopausal women	45–65	Brazil	Lumbar spine	DXA	International Society for Clinical Densitometry	N=36, 10 g daily collagen hydrolysate	Daily for 24 weeks	N=36, placebo: 10 g maltodextrin	None	Serum BALP, CTX, and OC
Daly 2020	January 2009 to May 2011	ACTRN12609000100291	RCT	Community-dwelling independently living men and women	≥ 60	Australia	Total hip and spine	DXA	BMD T-score between -1.0 and -2.5 SD	N=77, multicomponent exercise, osteoporosis education, and theory-based behavioral change program. A 12-month supervised and structured program, a 6-month “research-to-practice” translational phase	Three nonconsecutive days per week (~60 minutes sessions) for 18 months	N=71, continue their usual self-care and were provided with general consumer material available from Osteoporosis Australia about osteoporosis	1000 IU of vitamin D and 700 mg of elemental calcium as calcium phosphate daily.	Total hip, FN, and lumbar spine BMD, trabecular bone volume fraction, number, thickness, and separation for distal femur and proximal tibia
Elam 2015	NA	NA	Double-blind RCT	Postmenopausal women	55.7 ± 3.3	USA	Lumbar spine, total body, and total hip	DXA	BMD T-score between -1.5 and -2.5	N=19, 5 g daily calcium-collagen chelate dietary supplement	Daily for 12 months	N=19, placebo	500 mg elemental calcium and 200 IU of vitamin D daily	Whole body, total hip, and L1–L4 lumbar spine BMD, and serum BALP, TRACP5b
Eslamipour 2023	NA	NA	Single-blind RCT	Postmenopausal women	50–56	Iran	Lumbar (L1–L4 level) and FN	DXA	BMD T-score between -1.5 and -2.5	(1) N = 15, high intensity resistance training, started the program at 70% in the first 4 weeks in a low repetition (8 reps) and reached 85% 1-rep max; (2) N = 15, low-intensity resistance training, implemented 40% 1-rep max at the beginning of the program with a high repetition (16 reps) of 40% and reached 60% 1-rep max.	20–60 minutes, 3 times weekly for 24 weeks	N=15, continued daily activities	None	Lumbar spine BMC, BMD, T-score, and Z-score, FN BMC, BMD, T-score, and Z-score
Filip 2015	December 2008 to March 2010	NCT00789425	Double-blind RCT	Women at least 24 months after cessation of menses or oophorectomy	49–68	Poland	Lumbar spine (L2–L4)	DXA	BMD T-score between -1.5 and -2.5	N=27, daily 250 mg of an olive-leaf extract, containing a minimum of 40% olive polyphenols	Daily for 12 months	N=21, placebo	400 mg elemental calcium daily.	Serum ALP, calcium, 25-OH-D, phosphate, hs-CRP, IL-6, OC/CTX
Granchi 2018	January 2016 to June 2017	NCT02731820	Double-blind RCT	Postmenopausal women	NA	Italy	Lumbar (L2–L4 level) and femoral	DXA	BMD T-score between -1.5 and -2.5	N=20, 30 mEq K citrate daily	Daily for 6 months	N=20, placebo	400 IU vitamin D3 cholecalciferol and 500 mg calcium carbonate daily	Serum CTX, BALP, P1NP, TRACP5b, PTH
Hooshmand 2016	August 2012 and September 2013	NCT02325895	RCT	Postmenopausal women	65–79	USA	Whole-body, lumbar spine, total hip, and forearm	DXA	BMD T-score between -1.0 and -2.5	(1) N = 16, 50 g/day of dried plum; (2) N = 13, 100 g/day of dried plum	Daily for 6 months	N=13, control	500 mg calcium carbonate plus 400 IU vitamin D3 daily	Serum BALP, TRACP-5b, BALP/TRACP-5b ratio, hs-CRP, IGF-1, RANKL, OPG, RANKL/OPG ratio, 25-OH-D, calcium, phosphorous
Jafarnejad 2017	May 2016 to October 2016	IRCT2015092024103N1	Double-blind RCT	Postmenopausal women	50–72	Iran	NA	DXA	BMD T-score between -1.5 and -2.5	N=20, multispecies probiotic supplement (capsule) daily	Daily for 6 months	N=21, placebo	500 mg Ca plus 200 IU vitamin D daily.	Spinal and total hip BMD, serum BALP, CTX, RANKL, OC, RANKL/OC ratio, DPD, OPG, TNF-a, IL-1, Vitamin D, PTH, calcium, phosphorus, magnesium, ALP
Kemmler 2004	NA	NA	RCT	Postmenopausal women	48–60	German	lumbar spine or total hip	DXA	BMD T-score between -1.0 and -2.5 SD	N=50, supervised group sessions (warm-up/ endurance, jumping, strength, and flexibility sequences) for 60–70 minutes, and non-supervised individual home training sessions (isometric, belt, and stretching exercises.) for 25 minutes.	4 sessions weekly for 26 months	N=33, continued habitual lifestyle	calcium and cholecalciferol supplementation according to their nutritional intake	Change percentage for lumbar spine, total hip, FN, trochanter, intertrochanter, total forearm, and ultradistal radius BMD, pain frequency and intensity
Kim 2019	NA	NA	RCT	Old women	65 or older	Korea	NA	DXA	BMD T-score between -1.5 and -2.5	N=9, circuit training 3 times weekly, warm-up and warm-down, 12 to 15 times for 40 sec each time: Dumbbell Press, Dumbbell Lateral Raise, Dumbbell Curl, Dumbbell Overhead Triceps Extension, Push Up, Crunch, Back Extension, Lunge, and Squat	3 times weekly for 8 weeks	N=9, control	None	Serum OC, T-score of BMD (site was not specified)
Lambert 2017	2013 to February 2015	NCT02174666	Double-blind RCT	Postmenopausal women	60–85	Denmark	NA	DXA	BMD T-score between -1.5 and -2.5	N=38, red clover extract (Combined total of 60 mg bioavailable isoflavones and probiotics) daily	Twice daily 12 months	N=40, placebo: 90 L of water mixed with 250 g brown food coloring	Tablets containing 1040 mg Ca, 487 mg Mg, and 25 mg vitamin D daily	Serum CTX, P1NP, OC, RANKL, OC, undercarboxylated OPG, estradiol
Lecomte 2023	August 2019 to December 2020	NCT04004013	Double-blind RCT	Postmenopausal women	50–85	Ireland	Any site	DXA	BMD T-score between -1.0 and -2.5	N=50, 1 capsule containing hop extract daily	Daily for 48 weeks	N=50, placebo	Two capsules containing 1000 mg calcium and 400 IU vitamin D3, daily	BMD
Li 2023	NA	ChiCTR2000039049	RCT	Postmenopausal women	50–70	China	NA	DXA	BMD T-score between -1.5 and -2.5	N=16, 30-minute progressive-intensity Yi Jin Jing plus elastic band resistance exercise three times weekly	3 times weekly for 6 months	N=14, maintained their lifestyle behaviors (diet and exercise habits) unaltered	None	Whole body, upper limbs, thighs, trunk, pelvis, spine, lumbar, BMD, serum OC, 1,25-(OH)2-D3, and calcium
Mainini 2012	NA	NA	RCT	Consecutive osteopenic postmenopausal women	≥ 45	Italy	NA	Quantitative ultrasonometry	BMD T-score between -1.0 and -2.5	N=23, oral tablets containing 300 mg alpha linolenic acid, 30 mg vitamin C, 5 mg vitamin E, and 2.75 mg selenium twice daily	Twice daily for 12 months	N=21, placebo: plant fiber.	Oral tablets containing 500 mg calcium and 400 IU vitamin D3, twice daily	Heel BMD
Maria 2017	NA	NA	Double-blind RCT	Postmenopausal women	49–75	USA	Left FN, total left hip, and lumbar spine L1-L4	DXA	BMD T-score between -1.5 and -2.5	N=11, MSDK: 5 mg melatonin, 450 mg strontium (citrate), 2000 IU vitamin D3 and 60 μg vitamin daily K_2_, divided into two capsules	Daily for 12 months	N=11, placebo: plant fiber.	None	BMD T-score of total left hip, lumbar spine L1-L4, achilles heel
Norton 2022	October 2018 to July 2019	NCT03701113	Block RCT	Postmenopausal women	50–70	Ireland	AP spine (L1–L4)	DXA	BMD T-score between -1.5 and -2.5	N=32, milk-based protein supplement comprised 0.3 g/kg body mass protein and 0.3 g/kg body mass carbohydrate daily	Daily for 24 weeks	N=35, control: an equivalent amount of carbohydrate energy, i.e., 0.6 g/kg body mass	None	Serum CTX, P1NP, spine, dual femur BMD
Rønn 2021	August 2013 to May 2014	NA	RCT	Postmenopausal women	67.3 ± 4.4	Denmark	Lumbar spine (L1-L4) and left or right hip	DXA	NA	N=62, 375 μg MK-7 daily	Daily for 3 years	N=57, identical placebo tablet	800 mg calcium and 38 μg vitamin D daily	BMD at total hip, FN, spine, tibia, radius, tibia cortical, tibia trabecular, radius cortical, radius trabecular, trabecular number, thickness, and spacing for distal tibia and radius, serum 25-OH-D, P1NP, CTX, BALP, OC
Sales 2020	November 2011 and December 2017	NA	Double-blind RCT	Postmenopausal women	≤ 70	Brazil	Lumbar spine, FN or total hip	DXA	BMD T-score between -1.5 and -2.5	N=106, 3 g creatine monohydrate daily	Daily for 24 months	N=94, placebo: dextrose	None	BMD and its T-score at whole body, lumbar spine, FN, total hip, trabecular number, thickness, and separation for tibia and radius
Shen 2012	NA	NA	Stratified RCT	Postmenopausal women	Green tea polyphenols (GTP): 56.5 ± 5.5, Tai Chi (TC): 58.3 ± 7.7, GTP+TC: 57.6 ± 6.7, placebo: 57.6 ± 7.5,	USA	Lumbar spine and/or hip	DXA	BMD T-score between -1.0 and -2.5 SD	(1) N = 39, Green tea polyphenols (GTP) 500 mg daily; (2) N = 37, TC: medicinal starch 500 mg daily and 24-move simplified Yang-style Tai Chi training (60 minutes per session, 3 sessions per week); (3) N = 37, GTP + TC: GTP 500 mg daily and 24-move simplified Yang-style TC training (60 minutes per session, 3 sessions per week)	6 months	N=37, placebo: medicinal starch 500 mg daily	500 mg elemental calcium and 200 IU vitamin D daily	Serum BALP, PTH, TRACP
Vallibhakara 2021	May 2018 to May 2019	NA	Double-blind RCT	Postmenopausal women	Over 45	Thailand	Lumbar spine, total hip or FN	DXA	BMD T-score between -1.5 and -2.5	N=26, 400 IU mixed-tocopherol daily	Daily for 12 weeks	N=26, placebo: soybean oil	Calcium and 200 IU vitamin D daily	Serum CTX and P1NP
Wen 2017	NA	NA	RCT	Postmenopausal women	58.2 ± 3.5	China	Lumbar spine	DXA	BMD T-score <-1.0, with baseline as −2.00 ± 0.67	N=24, 1.5-hour short-term group-based step aerobics for three times per week: a 10 to 15- min warm-up, a 35 to 45-min step aerobics exercise, a 10 to 15- min balance and cool-down session, and a 10 to 15-min stretching and relaxation session	Three times per week for ten weeks	N=22, control	None	BMD, BMC, and BA at whole body and total hip, Serum OC, CTX, OC/CTX ratio, and function fitness

a All the participants in all arms received these treatments. Abbreviations: ALP, alkaline phosphatase; BA, bone area; BALP, bone-specific alkaline phosphatase; BMC, body mineral content; BMD: body mineral density; CTX, C-terminal telopeptide of collagen; BMR, Basal metabolic rate; DXA, dual energy X-ray absorptiometry; FN, femoral neck; hs-CRP, high-sensitivity C-reactive protein; IGF-1, insulin-like growth factor-1; IL-1, interleukin 1; IL-6, interleukin 6; NA, not available; NTX, N-telopeptides of type I collagen; OC, osteocalcin; OPG, osteoprotegerin; PTH, parathyroid hormone; P1NP, procollagen type I N-terminal propeptide; RANKL, receptor activator of nuclear factor kappa-B ligand; RCT, randomized controlled trial; TBS, trabecular bone score; TC, Tai Chi; TNF-α, tumor necrosis factor; TRACP, tartrate-resistant acid phosphatase; 25-OH-D, 25 hydroxyvitamin D.

### 3.2 Quality assessments of included articles

**Figure 2:**
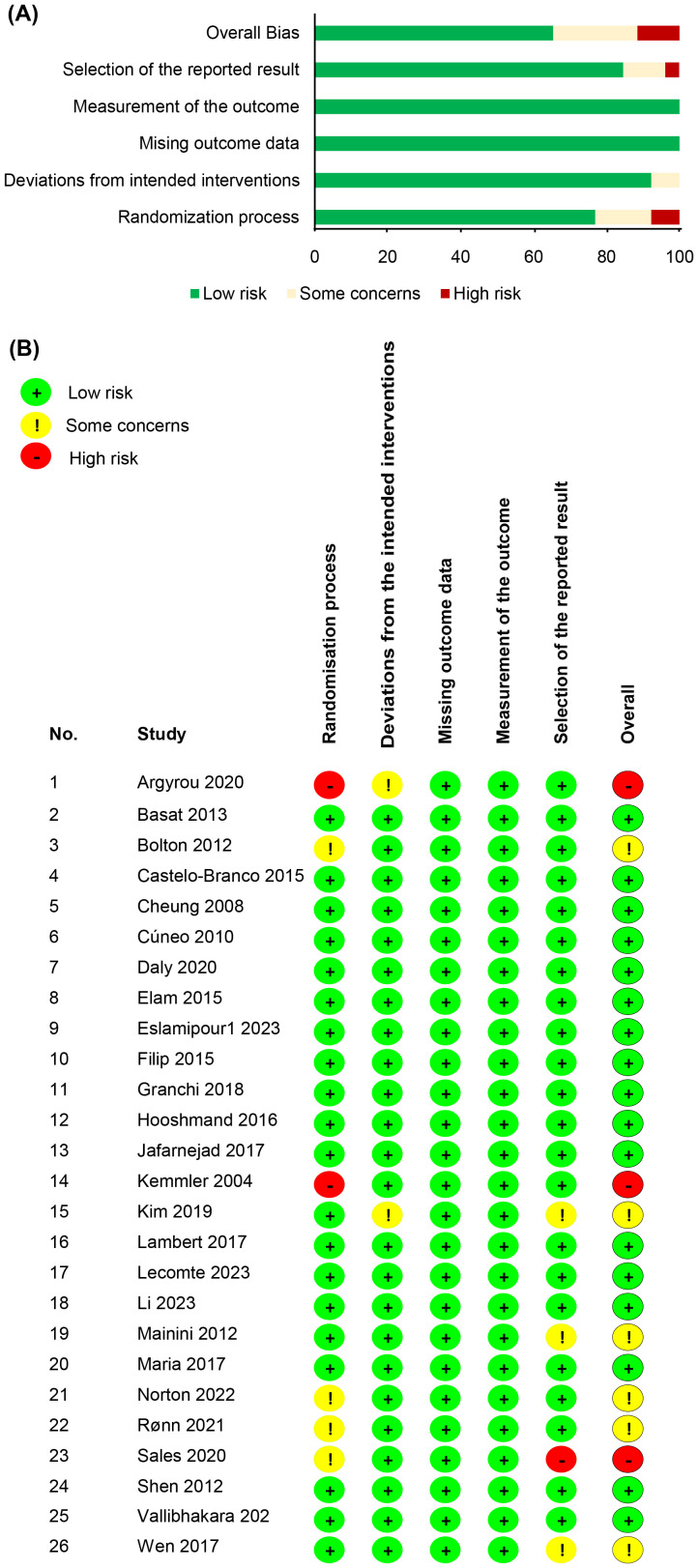
Results of quality assessment for **(A)** all the studies and **(B)** each study.

Quality assessment results (ROB 2) are shown in **Figure 2**. Seventeen were rated as low risk of bias, 6 had some concerns, and 3 as high risk of bias, based on the ROB 2 tool.

### 3.3 Categorization of evidence levels and statistical analysis

#### 3.3.1 Exercise

Nine studies examined exercise interventions, including single exercise types (e.g. resistance training, step aerobics, and Tai Chi) and multicomponent programs combining various exercise types (28, 29, 33, 39, 42, 48, 50, 52, 53). Four studies reported to follow American College of Sports Medicine (ACSM) guidelines (28, 33, 39, 50), and one adhered to exercise recommendations for postmenopausal women with osteoporosis (42), including parameters of frequency, intensity, and duration. Other studies, while not explicitly citing formal guidelines, described protocols with appropriate loading parameters (e.g., % 1RM resistance) or referenced supporting literature (29, 48, 52, 53). The intervention frequency and duration were similar, with sessions typically lasting 60 to 90 minutes. This duration included a warm-up period of 5 to 15 minutes, followed by a training session lasting 45 to 80 minutes. The interventions were typically conducted three times per week. However, the duration of the intervention periods varied across the studies, ranging from 8 weeks to 52 weeks.

Fewer than three trials examined any single exercise modality for a given outcome, limiting our ability to assess modality-specific effects. However, bone adaption is site-specific, meta-analyses were conducted on studies reporting outcomes at the same skeletal site.

Interventions using single exercise types aimed to increase muscle strength through activities such as isometrics, push-ups, and squats (29, 52), improve endurance through aerobic exercises like step aerobics and jump rope (29, 50), or enhance mind-body coordination through Tai Chi (48). Multicomponent programs incorporated various types of exercises and psychological elements, and repeating load bearing exercise through circuit training (39). Others combined a traditional Chinese medicine exercise practice known as Yi Jin Jing with elastic band resistance exercise, as well as a combination of resistance training, impact loading, and balance exercises (29, 33, 39, 42, 53).

##### 3.3.1.1 Meta-analysis

**Figure 3:**
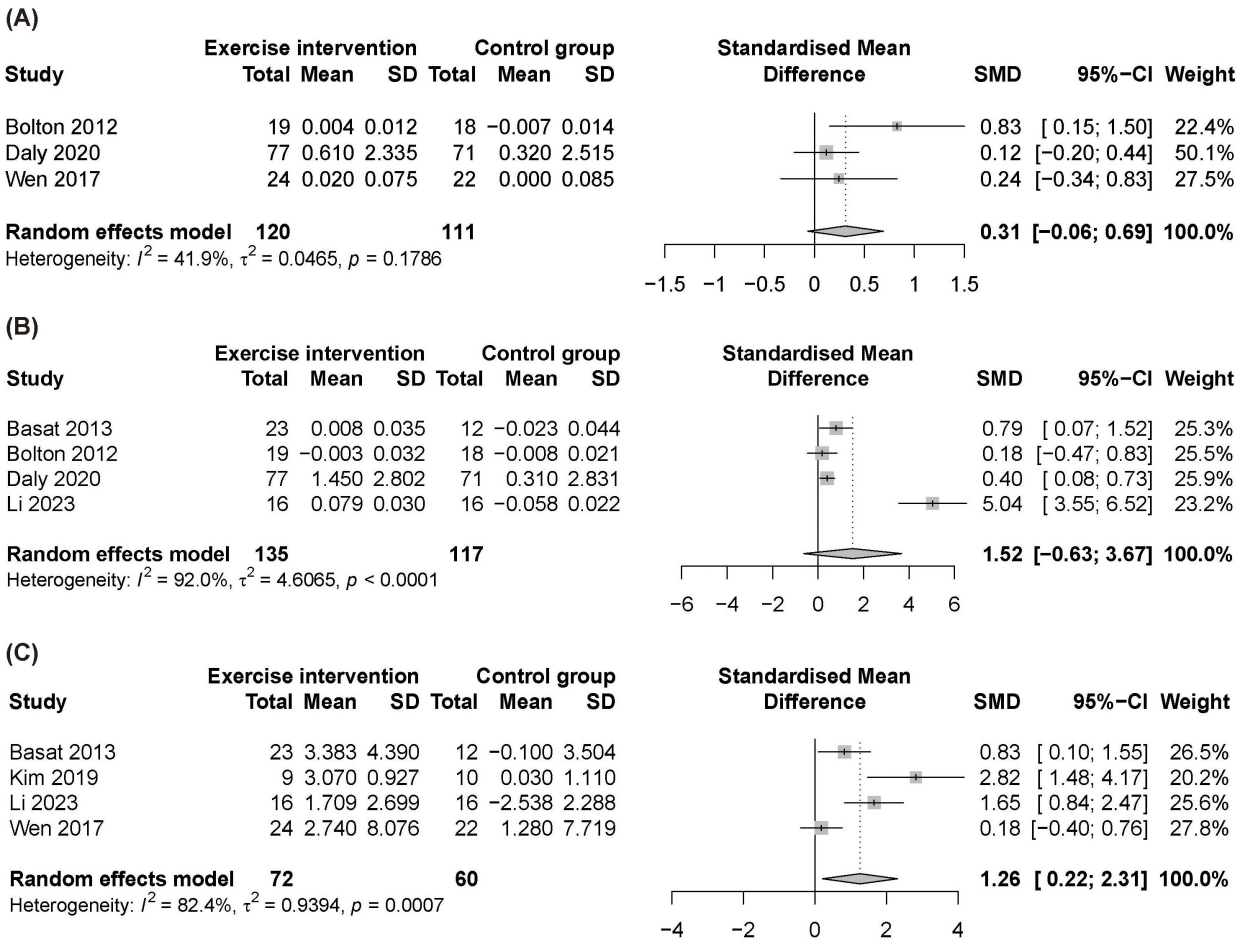
Forest plot of comparing **(A)** total hip bone mineral density, **(B)** lumbar spine bone mineral density, and **(C)** osteocalcin between exercise intervention and control group. CI, confidence interval; MD, mean difference; SD, standard deviation; SMD, standardized mean difference.

**Figure 3** shows the results of the meta-analysis for total hip BMD, lumbar spine BMD, and OC, respectively. Increased serum OC was also observed (SMD = 1.26, 95% CI: 0.22–2.31). The intervention included a mixture of multicomponent programs (isometric strengthening and high-impact exercises, Yi Jin Jing plus elastic band resistance exercise, and full-body strength training combination) and jump rope (28, 39, 42, 50). However, no significant difference was found for total hip and lumbar spine BMD. Egger’s test did not indicate small-study effects for lumbar spine (*P* = 0.365) or total hip (*P* = 0.233); the test for OC was borderline (*P* = 0.051). FN BMD data were insufficient for meta-analysis.

##### 3.3.1.2 Narrative synthesis

The evidence categorization presented in **Table 3** indicated a positive effect of exercise interventions on lumbar spine and FN BMD, as well as increased OC. No significant effects were observed on whole body BMD, whole body BMC, and the impact on other outcomes (total hip BMD, lower limb and total hip BMC, BALP CTX, NTX, PTH, BAP/TRAP5b, and 25-OH-D3) could not be determined due to the limited data.

**Table 3:** Level of evidence examining the effects of different non-pharmacological interventions.

Intervention	Outcome	No. of studies	No. of studies with positive effects	No. of studies with no effects	No. of studies with negative effects	Overall effects
Exercise	BMD					
	Whole body BMD	3 (29, 42, 50)	1 (42)	2 (29, 50)	0	0
	Lumbar spine BMD	6 (28, 29, 33, 42, 52, 53)	5 (28, 33, 42, 52, 53)	1 (29)	0	**+**
	Total hip BMD	4 (29, 33, 50, 53)	2 (29, 53)	2 (33, 50)	0	?
	FN BMD	4 (28, 33, 52, 53)	4 (28, 33, 52, 53)	0	0	**+**
	BMC					
	Whole body BMC	2 (29, 50)	0	2 (29, 50)	0	0
	Lower limb BMC	1 (29)	0	1 (29)	0	?
	Total hip BMC	1 (50)	0	1 (50)	0	?
	Bone formation markers					
	BALP	1 (48)	1 (48)	0	0	?
	OC	3 (28, 39, 50)	2 (28, 39)	1 (50)	0	**+**
	Resorption markers					
	CTX	1 (50)	0	1 (50)	0	?
	NTX	1 (28)	1 (28)	0	0	?
	PTH	1 (48)	1 (48)	0	0	?
	Balance					
	BAP/TRAP5b	1 (48)	1 (48)	0	0	?
	25-OH-D_3_	1 (42)	1 (42)	0	0	?
Vitamin K	BMD					
	Lumbar spine BMD	1 (31)	0	1 (31)	0	?
	Total ip BMD	2 (31, 46)	0	2 (31, 46)	0	0
	FN BMD	2 (31, 46)	1 (31)	1 (46)	0	?
	ultradistal radius BMD	1 (31)	0	1 (31)	0	?
	Bone formation markers					
	BALP	1 (46)	0	1 (46)	0	?
	OC	2 (31, 46)	0	0	2 (31, 46)	**-**
	ucOC	2 (31, 46)	2 (31, 46)	0	0	+
	P1NP	1 (46)	0	1 (46)	0	?
	Resorption markers					
	CTX	2 (31, 46)	0	2 (31, 46)	0	0
MMN	BMD					
	Lumbar spine BMD	1 (44)	1 (44)	0	0	?
	Total hip BMD	1 (44)	0	1 (44)	0	?
	FN BMD	1 (44)	1 (44)	0	0	?
	Heel BMD	2 (43, 44)	1 (43)	1 (44)	0	?
	Bone formation markers					
	OC	1 (44)	0	1 (44)	0	?
	P1NP	1 (44)	1 (44)	0	0	?
	Resorption markers					
	CTX	1 (44)	0	1 (44)	0	?
Collagen supplements	BMD					
	Whole body BMD	1 (34)	0	1 (34)	0	?
	Lumbar spine BMD	1 (34)	0	1 (34)	0	?
	Total hip BMD	1 (34)	1 (34)	0	0	?
	Bone formation markers					
	BALP	2 (32, 34)	0	2 (32, 34)	0	0
	OC	2 (32, 34)	0	2 (32, 34)	0	0
	P1NP	1 (27)	0	0	1 (27)	?
	Resorption markers					
	TRACP5b	1 (34)	0	1 (34)	0	?
	CTX	2 (27, 32)	0	1 (32)	1 (27)	?
	Balance					
	BAP/TRAP5b	1 (34)	1 (34)	0	0	?
Polyphenol extracts	BMD					
	Lumbar spine BMD	2 (35, 40)	2 (35, 40)	0	0	**+**
	FN BMD	3 (35, 40, 41)	1 (40)	2 (35, 52)	0	0
	Bone formation markers					
	BALP	1 (48)	1 (48)	0	0	?
	OC	1 (40)	0	1 (40)	0	?
	ucOC	1 (40)	0	1 (40)	0	?
	P1NP	1 (40)	0	1 (40)	0	?
	OPG	1 (40)	0	1 (40)	0	?
	Resorption markers					
	CTX	1 (40)	1 (40)	0	0	?
	PTH	1 (48)	0	1 (48)	0	?
	Balance					
	BAP/TRAP5b	1 (48)	1 (48)	0	0	?
	Hormones and cytokines					
	Estradiol	1 (40)	0	1 (40)	0	?
	IL-6	1 (35)	0	1 (35)	0	?

Overall effects of the intervention: +, positive; -, negative; 0, no effect;?, inconsistent. Abbreviations: BALP, bone-specific alkaline phosphatase; BMC, body mineral content; BMD: body mineral density; CTX, C-terminal telopeptide of collagen; FN, femoral neck; IL-1, interleukin 1; IL-6, interleukin 6; MMN, multiple micronutrients; OC, osteocalcin; OPG, osteoprotegerin; PTH, parathyroid hormone; P1NP, procollagen type I N-terminal propeptide; TNF-α, tumor necrosis factor; TRACP, tartrate-resistant acid phosphatase; ucOC, uncarboxylated osteocalcin; 25-OH-D_3_, 25 hydroxyvitamin D_3_.

Single-mode exercise interventions demonstrated a positive impact on lumbar spine and FN BMD. However, the effects on BMD for other sites and BTMs varied (28, 48, 50, 52). Multicomponent exercise interventions demonstrated positive effect on FN BMD (29, 33, 39, 42, 53).

#### 3.3.2 Nutrition intervention

A total of 18 studies investigated the effect of dietary or nutrition interventions, including micronutrient supplements (six studies), collagen or milk-derived protein matrix supplements (five studies), polyphenol extracts (four studies), dried plum (one study), probiotic supplement (one study), and creatine (one study). The intervention frequency was consistent across the studies, typically on a daily basis. However, the duration of the interventions varied, ranging from 12 weeks to 3 years. Most studies provided both intervention and placebo groups with calcium and vitamin D to meet essential nutrient needs.

##### 3.3.2.1 Micronutrient supplements or fortified foods

Both single and multiple micronutrients (MMN) were tested. The most consistent finding was a decrease in undercarboxylated osteocalcin (ucOC) (**Table 3**). Two studies investigated the effects of vitamin K supplementation with one using vitamin K1 (31) and the other with vitamin K2 (46). For both studies, the effects of vitamin K on other outcomes could not be determined due to limited number of studies. Other single nutrients (potassium citrate, vitamin E) showed no BMD/BTM changes, except vitamin E reduced CTX (31, 46).

Two studies examined the effects of MMN. One study involved a combination of vitamin D3, vitamin K2, melatonin, and citrate, while the other study used a combination of vitamin C, vitamin E, selenium, and alpha-lipoic acid. Although both studies showed some benefits for bone health outcomes, the heterogeneous outcomes precluded firm conclusions (43, 44).

##### 3.3.2.2 Collagen or other proteins supplements

Five studies investigated the effectiveness of protein supplements, with four using collagen supplements, reporting inconsistent findings, as shown in **Table 3**. Among them, two with calcium-collagen chelate or ossein hydroxyapatite and calcium carbonate complex improved whole BMD and pain (30, 34). However, these effects were not observed in the other two RCTs with collagen hydrolysate or collagen peptide (27, 32). Another RCT reported a calcium-fortified, milk-derived protein matrix increased P1NP levels and reduced CTX levels, while showing no effects on BMD or other serum markers (45).

##### 3.3.2.3 Polyphenol extracts

Three studies examined the effects of polyphenol extracts from different sources, including red clover, olive, and green tea. As shown in **Table 3**, they all reported significant effects on improving lumbar spine BMD (35, 40, 48), but effects on other outcomes were inconsistent.

##### 3.3.2.4 Food interventions

We only identified one study that investigated the effects of dried plum on bone health in the US (37). The study demonstrated a beneficial effect in improving several BTMs and bone BMD but not for other markers.

##### 3.3.2.5 Other nutrition interventions

Other nutrition interventions included probiotics and creatine. One RCT reported that the probiotic supplement reduced BALP, CTX, and PTH but did not affect BMD or other BTMs (38). Another RCT with creatine supplementation showed no significant effects on BMD, microarchitecture parameters, or BTMs (47).

**Figure 4:**
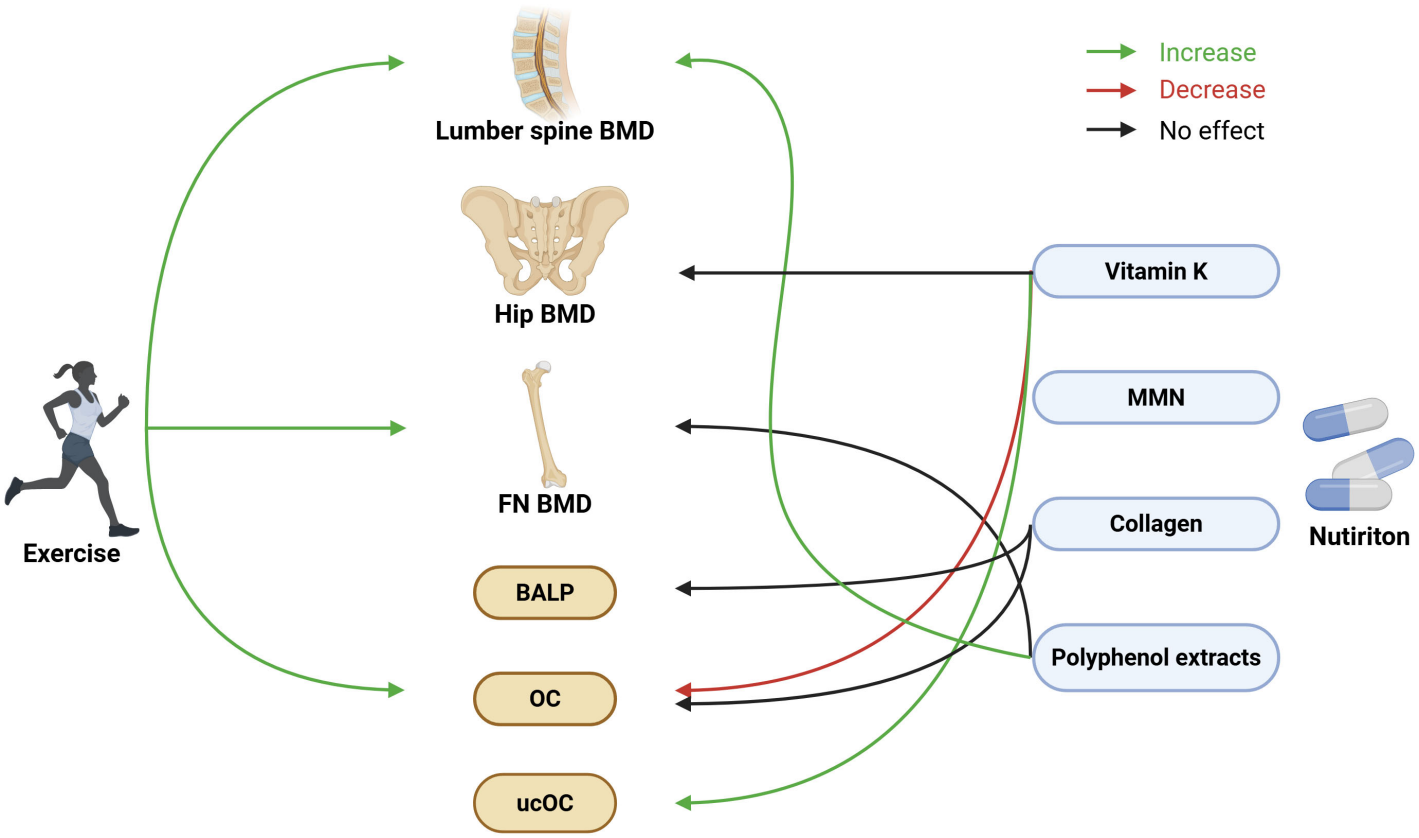
Summary of effect of non-pharmacological interventions on bone health among patients with low bone mass. Created in BioRender. ZHANG, J. (2025) https://BioRender.com/01390wd.

The summary of the results is shown in **Figure 4**.

## 4 Discussion

This systematic review and meta-analysis identified twenty-six non-pharmacological intervention studies focused on individuals with low bone mass. In this review, low bone mass was defined as a T-score between −1.0 and −2.5, based on the diagnostic criteria established by the World Health Organization expert working group (54). Our analysis suggested that exercise interventions led to a significant increase in lumbar spine and FN BMD as well as OC levels in participants with low bone mass. In terms of nutrition interventions, we found that polyphenol extracts from various plant sources showed effectiveness in improving lumbar spine BMD. However, the effects of other nutrition interventions reviewed were found to be limited.

### 4.1 Exercise and low bone mass

Physical exercise is the most effective non-pharmaceutical fracture prevention strategy (55). However, the precise mechanisms through which exercise impacts bone health are not yet fully understood. Exercise affects bone health by influencing apoptosis, inflammatory response, and autophagy (56). It may also affect the epigenetic mechanisms of bone metabolism by regulating non-coding RNAs and DNA methylation (56). Exercise may also increase serum vitamin D levels and affect BTMs (57). According to our synthesis of studies, exercise intervention programs designed with frequency, intensity and time parameters in line with established exercise guidelines for bone health—such as those from the ACSM or recommendations for postmenopausal women—resulted in increased lumbar spine and FN BMD. However, we did not observe similar effects on BMD at other sites. These findings align with previous reviews conducted in individuals aged 65 years and above (58, 59) as well as in patients with osteoporosis and low bone mass (58, 59).

Furthermore, our study showed that exercise increased OC, but not other BTMs. While we did not find similar reviews specific to population with low bone mass, previous reviews in the general population have demonstrated effects of acute exercise on various BTMs, including increased OC (60, 61). It is important to note that some studies have observed a negative association between OC level and BMD (62). As exercise can influence multiple functions of OC (60), OC alone may not be a reliable marker of high bone turnover status in postmenopausal osteoporosis, considering the fact that changes in OC levels may not solely reflect alterations in bone metabolism (63). Of note, Egger’s test yielded a borderline result (*P* = 0.051), suggesting a possible risk of publication bias that should be interpreted with caution. Therefore, the significance of the increase in OC through exercises and its relation to long-term outcomes in population with low bone mass need further verification.

Notably, according to the studies, half of the exercise protocols were reported to meet recommendations for bone health in adults by ACSM, typically involving 60- to 90-min sessions for three times a week (64, 65). Moreover, although the two included short-term studies may limit the interpretation of long-term effects (shorter than three months), one of them still demonstrated measurable improvements in bone health. The role of exercise in maintaining or increasing BMD remains inconclusive. Nevertheless, even older individuals with frailty are advised to remain physically active according to standard exercise recommendations, due to the rapid and profound effects of immobilization on low bone mass and the poor prognosis for mineral recovery after remobilization (66). Notably, safety considerations should be taken into account, and the type, frequency, and duration of exercise may need to be adjusted (67). For example, both higher-impact activities and resistance exercises with higher impact have demonstrated greater benefits for bone health compared to lower impact sports, but individual responses to exercise can vary, leading to the conservative prescription of training loads to balance efficacy and safety.

The effects of different exercise interventions on BMD vary depending on whether single type or multicomponent exercises are utilized, but the limited number of studies examining each type of intervention, particularly for single type exercises, hinders a comprehensive evaluation of their diverse impacts on specific outcomes in population with low bone mass. Current guidelines for osteoporosis prevention and treatment recommend weight-bearing exercises for BMD benefits, while strengthening exercises and balance training may help maintain bone mass and prevent of fall-related fractures. A meta-analysis suggests that combining different exercises in postmenopausal women appears to be effective in preserving BMD at various skeletal sites (68). This is believed to be achieved by generating diverse mechanical strains and impacting different loading areas of the bones.

Therefore, future research should prioritize investigating the minimum effective dosage and impact of different exercise types, including the combined effect of diverse exercises, as an exercise intervention for low bone mass. Such investigations will enhance our understanding of the specific role of exercise in managing low bone mass and improving bone health.

### 4.2 Nutrition intervention and low bone mass

Previous meta-analyses and clinical guidelines have already established that individuals with osteoporosis should receive calcium and vitamin D supplementation to reduce fracture risk and improve bone health (3, 69); therefore, none of the included studies specifically examined these two nutrients as standalone interventions. Our review identified nutrition interventions that go beyond calcium and vitamin D with the aim of enhancing bone health outcomes in population with low bone mass. These interventions include micronutrients, collagen supplementation, polyphenol extracts, and other nutrition solutions such as probiotics, dried plum, creatine, and milk-derived protein matrix fortified with calcium. The rationale behind these interventions is to improve calcium absorption efficiency, promote bone metabolism, and provide antioxidant properties to individuals with low bone mass.

However, the number of studies investigating similar nutrition interventions was limited, which hindered the possibility of conducting a meta-analysis to evaluate effect of interventions on bone health outcome. The level of evidence evaluation yielded a mixed result.

#### 4.2.1 Vitamin K and multiple micronutrient supplementations

The studies in this review included the effects of vitamin K supplementation and the use of a multi-micronutrient approach.

For vitamin K, two RCTs showed that vitamin K had no significant effect on BMD for population with low bone mass. Vitamin K is recognized as an essential nutrient for bone health as it participates in carboxylation of bone-related proteins, regulates the genetic transcription of osteoblastic markers, and helps regulate bone reabsorption (70, 71). However, different reviews examining the relationship between vitamin K supplementation and bone health outcomes have reported inconsistent conclusions due to heterogeneity across studies (72, 73). A subgroup analysis from the meta-analysis conducted by Huang et al. indicated a significant improvement in vertebral BMD for postmenopausal women with osteoporosis in the vitamin K2 group, but no significant difference in BMD changes was observed in the non-osteoporosis subgroup (74). The authors suggested that two potential reasons: 1) higher baseline BMD in non-osteoporotic participants yields smaller relative percent changes for the same absolute change; 2) greater baseline mineralization may limit additional mineral accrual. In this review, the two included studies utilized different forms of vitamin K, with only one study each, thus limiting the ability to reach conclusions.

Moreover, vitamin K supplements decreased total OC level, which differed from previous studies showing that vitamin K increased OC and decreased ucOC (75, 76). The decreased OC observed in our study might be explained by two hypotheses: (1) improved vitamin K status leads to more OC being carboxylated to its bioactive form, resulting in less OC being needed, synthesized, and released into the circulation (31); (2) more functional OC will be bound to bone rather than circulating in the blood stream, thereby lowering the serum total OC level (77). However, it should be noted that the included studies exhibited methodological heterogeneity, with variations in the type of vitamin K supplements (vitamin K_1_ and K_2_) which have different bioavailability and half-lives (78). Therefore, for population with low bone mass, further studies are needed to draw conclusive evidence on the effects of vitamin K combined with vitamin D or calcium on bone health outcome, as it has been suggested that this combination may have a greater synergistic effect in reducing low bone mass (79, 80).

For MMN, two studies were included with both reporting observed favorable effects on bone health. However, due to the limited number of only 2 studies available using different MMN combination, we were unable to draw a definitive conclusion. Nevertheless, existing evidence has shown that micronutrients such as calcium, vitamin D, vitamin C, and vitamin E play important roles in preventing low bone mass (81, 82). Although there has not been a comprehensive review on the relationship between MMN supplements and bone health, increasing evidence suggests that through synergistic effect, combined effects of two or more nutrients working together have a greater physiological impact on the body than when each nutrient is consumed individually (83). Therefore, to further examine the health effects of MMN on low bone mass, studies with a clear mechanism-driven approach that target the combined use of different micronutrients for population with low bone mass are needed.

#### 4.2.2 Collagen supplementations

Collagen, a major component of the organic bone matrix, may promote osteoblastic cell growth and differentiation while reducing osteoclastic activity, thereby supporting bone formation and mineralization. It increases osteoblastic cell growth and differentiation while reducing osteoclastic cells (84). Additionally, collagen may play a role in downregulating the production of pro-inflammatory molecules implicated in osteoporosis and low bone mass development (85).

In our study, the results for dietary collagen supplementation on bone health outcome in women with low bone mass were mixed. Few reviews have examined the effects of collagen or calcium-collagen chelate in patients with osteoporosis, with only one systematic review published in 2016 concluded that collagen might have a positive effect on osteoporosis, based only on two animal experiments (84).

The mixed results observed in our study can be attributed to several factors. Firstly, the efficacy of bone protection is influenced by the structure and quantity of peptides derived from collagen (84). Included interventions comprised collagen hydrolysates and calcium-collagen chelates, which may differ in digestion, absorption and bioavailability. Additionally, the calcium status of the subjects may have an impact on calcium retention and bone resorption, which could further contribute to the variability in the results (32). Therefore, further research direction should focus on the following aspects: (1) characterize the structure and number of peptides that relevant to low bone mass improvement; (2) considering calcium condition of the subjects when designing intervention research with collagen.

#### 4.2.3 Polyphenol extracts supplementations

In our study, the included RCTs using polyphenol extracts showed significant improvements in lumbar spine BMD but not in other outcomes of bone health. A recently published systematic review differed slightly from our results, indicating that polyphenol supplements increased BALP among postmenopausal women, while a significant effect on lumbar spine BMD only emerged for intervention with duration of lasting over 24 months (86). Trials used different types of polyphenols, which may exert distinct mechanisms relevant to bone health (87). Isoflavones, one of the most important categories of polyphenols, exert a pro-estrogenic activity on bone, and thereby inducing osteoclast apoptosis (88). Further exploration is needed to determine the effectiveness of different types of polyphenol extracts on bone health among individuals with low bone mass, using more robust evidence.

#### 4.2.4 Whole food supplementations (dried plum)

We found only one study involving a whole-food intervention with dried plums, or prunes, as intervention. Daily consumption of dried plum was reported to improve low bone mass in older, postmenopausal women with low bone mass (89). However, since there is only one RCT available, the evidence for its effect on low bone mass remains unverified.

Furthermore, while the role of foods such as dairy products in improving bone health has been well-established, which have found that dairy products, with or without vitamin D, increase BMD (90), further studies focusing on effect of whole food and dietary pattern as interventions may provide valuable evidence to investigate the potential benefits for population with low bone mass. These types of interventions are more likely to promote compliance and provide a comprehensive understanding of the impact of dietary patterns on bone health outcomes.

#### 4.2.5 Other nutrition supplementations

This review also identified other nutrition interventions, including one study on probiotics and another on creatine, for improving bone health in individuals with low bone mass. However, due to the limited number of studies available, no definitive conclusions can be drawn. Currently, there is a growing focus on researching the health benefits of probiotics, although their impact on population with low bone mass is less explored and merits further investigation.

### 4.3 Other research gaps on non-pharmacological intervention for low bone mass

This review has identified several research gaps regarding non-pharmacological interventions for low bone mass. Firstly, there is a research gap in the availability of comprehensive non-pharmacological interventions for low bone mass. Currently, only one RCT has been found that involves a combination of interventions targeting various lifestyle factors such as exercise, nutrition, smoking and drinking cessation, and health education. Comprehensive approach may have the potential to yield more significant effects for managing low bone mass (91). Therefore, further investigation is warranted to explore the effectiveness of comprehensive non-pharmacological therapies for low bone mass.

Secondly, the serum markers for bone turnover and resorption examined in the included studies may not be fully specific or sensitive for low bone mass, as they were primarily suitable for osteoporosis (92). BTMs, including OC, are influenced by factors such as circadian rhythm, dietary intake, comorbidities, and assay variability, which may limit their reliability as sole markers of intervention efficacy (93, 94). Furthermore, BTM changes often reflect short-term alterations in bone remodeling dynamics and may not directly correlate with long-term skeletal outcomes such as BMD gains or fracture reduction (95). Therefore, BTM results should be considered supportive rather than definitive evidence, and should be interpreted with caution. Moreover, the use of DXA to assess BMD was limited by asymptomatic nature of low bone mass (96). Other well-established clinical risk factors, such as fall history and composite risk assessment tools like the FRAX score, are recommended in clinical practice for fracture risk evaluation. However, their utility for guiding non-pharmacological interventions in populations with low bone mass remains unclear (97). Further investigation is needed to determine their utility in guiding and tailoring nonpharmacological interventions for this specific population.

Lastly, most studies included in the research focused on postmenopausal women, with only one study included both men and women. This is reasonable considering the higher prevalence of osteoporosis and related fractures in postmenopausal women, attributed to the pivotal role of estrogen in maintaining bone health (4). The decline in BMD begins with reduced estrogen levels around menopause and continues thereafter, as estrogen directly and indirectly influences bone by inhibiting bone resorption and promoting calcium excretion (98). Approximately half of women experience accelerated low bone mass, ranging from 10% to 20%, during the 5–6 years surrounding menopause (99). However, evidence suggests that the global male population also suffers from osteoporosis (11.7%) but is often not evaluated or treated in line with guidelines (4, 100). Considering the societal structure of an aging population, there is a need to investigate intervention strategies for older men with low bone mass. Further research should focus on addressing this gap.

### 4.4 Strengths and limitations

To the best of our knowledge, this study is the first systematic review and meta-analysis summarizing the efficacy of non-pharmacological interventions in population with low bone mass. However, this study has some limitations. Firstly, although we have searched literature comprehensively and examined the publication bias, this bias is still a potential limitation of systematic reviews. Secondly, most studies did not report medical treatment condition of the participants. Because pharmacotherapy is uncommon in low bone mass, unreported concomitant treatments could bias estimates and potentially underestimate or confound intervention effects. Third, because of the limited number of eligible studies, our analysis did not account for the specific skeletal sites targeted by each exercise intervention, which may have influenced the observed results. Finally, limited number of studies with scattered interventions and outcomes were included, contributing to high heterogeneity and underestimation of these interventions. The limited number of included studies, which prevents categorization of evidence levels and increases the susceptibility to publication bias. Future research, as more original studies become available, should aim to conduct meta-analyses to provide more precise and unbiased estimates.

## 5 Conclusion

In summary, addressing low bone mass is important for interventions aimed at improving bone health and preventing the associated morbidity and mortality from fractures. This has significant implications for public health. Low bone mass, similar to conditions like prediabetes, prehypertension, and borderline high cholesterol, represents an intermediate risk group with unclear boundaries. However, what makes low bone mass a critical phase is the large number of individuals affected by it, making this group a significant portion of the population at risk for fractures. This highlights the importance of targeting interventions towards individuals with low bone mass in order to effectively reduce the burden of fractures by improving bone health.

Non-pharmacological interventions, such as exercise and specific nutrition strategies, hold promise in maintaining bone health in individuals with low bone mass. In the included studies, exercise programs of approximately 60–90 minutes per session, performed three times per week and aligned with existing guidelines, were associated with modest BMD benefits at some skeletal sites, but we did not compare the effect size of different intervention parameter, and the parameter for other exercises warrants further investigation. Regarding nutrition interventions, polyphenol extracts showed efficacy on lumbar spine BMD, while the results of collagen supplements were mixed, and the effects of micronutrients supplements were limited.

However, it is important to acknowledge that these conclusions are preliminary due to the limited evidence currently available. More high-quality RCTs are needed to fill the research gap on comprehensive lifestyle interventions. Additionally, precise prevention strategies tailored for individuals in the lower range of low bone mass (e.g., using a T-score below -2.0 to identify those at higher risk of progressing to osteoporosis) should be further investigated. It is also important to evaluate the specific needs of older men, as interventions for this population are currently limited. Further research in these areas will enhance our understanding and enable the development of more evidence-based interventions for individuals with low bone mass.

## References

[1] Kanis JA Melton LJ 3rd Christiansen C Johnston CC Khaltaev N . The diagnosis of osteoporosis. J Bone mineral Res. (1994) 9:1137–41. doi: 10.1002/jbmr.5650090802, PMID: 7976495

[2] Gregson CL Armstrong DJ Bowden J Cooper C Edwards J Gittoes NJL . UK clinical guideline for the prevention and treatment of osteoporosis. Arch Osteoporos. (2022) 17:58. doi: 10.1007/s11657-022-01061-5, PMID: 35378630 PMC8979902

[3] LeBoff MS Greenspan SL Insogna KL Lewiecki EM Saag KG Singer AJ . The clinician’s guide to prevention and treatment of osteoporosis. Osteoporosis Int. (2022) 33:2049–102. doi: 10.1007/s00198-021-05900-y, PMID: 35478046 PMC9546973

[4] Salari N Ghasemi H Mohammadi L Behzadi MH Rabieenia E Shohaimi S . The global prevalence of osteoporosis in the world: a comprehensive systematic review and meta-analysis. J Orthop Surg Res. (2021) 16:609. doi: 10.1186/s13018-021-02772-0, PMID: 34657598 PMC8522202

[5] Klibanski A Adams-Campbell L Bassford T Blair SN Boden SD Dickersin K . Osteoporosis prevention, diagnosis, and therapy. J Am Med Assoc. (2001) 285:785–95. doi: 10.1001/jama.285.6.785, PMID: 11176917

[6] Lane NE . Epidemiology, etiology, and diagnosis of osteoporosis. Am J Obstetrics Gynecology. (2006) 194:S3–S11. doi: 10.1016/j.ajog.2005.08.047, PMID: 16448873

[7] Sanders KM Nicholson GC Watts JJ Pasco JA Henry MJ Kotowicz MA . Half the burden of fragility fractures in the community occur in women without osteoporosis. When is fracture prevention cost-effective? Bone. (2006) 38:694–700. doi: 10.1016/j.bone.2005.06.004, PMID: 16507356

[8] Varacallo M Seaman TJ Jandu JS Pizzutillo P . Osteopenia (2023). StatPearls Publishing. Available online at: https://www.ncbi.nlm.nih.gov/books/NBK499878/. 29763053

[9] Xiao PL Cui AY Hsu CJ Peng R Jiang N Xu XH . Global, regional prevalence, and risk factors of osteoporosis according to the World Health Organization diagnostic criteria: a systematic review and meta-analysis. Osteoporos Int. (2022) 33:2137–53. doi: 10.1007/s00198-022-06454-3, PMID: 35687123

[10] Roux C Briot K . Osteoporosis in 2017: Addressing the crisis in the treatment of osteoporosis. Nat Rev Rheumatol. (2018) 14:67–8. doi: 10.1038/nrrheum.2017.218, PMID: 29323345

[11] Kling JM Clarke BL Sandhu NP . Osteoporosis prevention, screening, and treatment: a review. J Womens Health (Larchmt). (2014) 23:563–72. doi: 10.1089/jwh.2013.4611, PMID: 24766381 PMC4089021

[12] Murad MH Drake MT Mullan RJ Mauck KF Stuart LM Lane MA . Comparative effectiveness of drug treatments to prevent fragility fractures: A systematic review and network meta-analysis. J Clin Endocrinol Metab. (2012) 97:1871–80. doi: 10.1210/jc.2011-3060, PMID: 22466336

[13] Alami S Hervouet L Poiraudeau S Briot K Roux C . Barriers to effective postmenopausal osteoporosis treatment: A qualitative study of patients’ and practitioners’ Views. PloS One. (2016) 11:e0158365. doi: 10.1371/journal.pone.0158365, PMID: 27355576 PMC4927112

[14] Zhu K Prince RL . Lifestyle and osteoporosis. Curr Osteoporosis Rep. (2015) 13:52–9. doi: 10.1007/s11914-014-0248-6, PMID: 25416958

[15] Martyanti RN Morikawa M Hanaoka M Tanaka S Nakamura Y Nose H . Increased response of postmenopausal bone to interval walking training depends on baseline bone mineral density. PloS One. (2024) 19:e0309936. doi: 10.1371/journal.pone.0309936, PMID: 39236022 PMC11376574

[16] Hejazi K Askari R Hofmeister M . Effects of physical exercise on bone mineral density in older postmenopausal women: a systematic review and meta-analysis of randomized controlled trials. Arch osteoporosis. (2022) 17:102. doi: 10.1007/s11657-022-01140-7, PMID: 35896850

[17] Qaseem A Hicks LA Etxeandia-Ikobaltzeta I Shamliyan T Cooney TG Cross JT Jr. . Pharmacologic treatment of primary osteoporosis or low bone mass to prevent fractures in adults: A living clinical guideline from the American college of physicians. Ann Internal Med. (2023) 176:224–38. doi: 10.7326/m22-1034, PMID: 36592456 PMC10885682

[18] Aibar-Almazán A Voltes-Martínez A Castellote-Caballero Y Afanador-Restrepo DF Carcelén-Fraile MDC López-Ruiz E . Current status of the diagnosis and management of osteoporosis. Int J Mol Sci. (2022) 23:9465. doi: 10.3390/ijms23169465, PMID: 36012730 PMC9408932

[19] Albrecht BM Stalling I Foettinger L Recke C Bammann K . Adherence to lifestyle recommendations for bone health in older adults with and without osteoporosis: cross-sectional results of the OUTDOOR ACTIVE study. Nutrients. (2022) 14:2463. doi: 10.3390/nu14122463, PMID: 35745193 PMC9228189

[20] Shams-White MM Chung M Du M Fu Z Insogna KL Karlsen MC . Dietary protein and bone health: a systematic review and meta-analysis from the National Osteoporosis Foundation. Am J Clin Nutr. (2017) 105:1528–43. doi: 10.3945/ajcn.116.145110, PMID: 28404575

[21] Xiaoya L Junpeng Z Li X Haoyang Z Xueying F Yu W . Effect of different types of exercise on bone mineral density in postmenopausal women: a systematic review and network meta-analysis. Sci Rep. (2025) 15:11740. doi: 10.1038/s41598-025-94510-3, PMID: 40188285 PMC11972399

[22] Moher D Liberati A Tetzlaff J Altman DG . Preferred reporting items for systematic reviews and meta-analyses: the PRISMA statement. PloS Med. (2009) 6:e1000097. doi: 10.1371/journal.pmed.1000097, PMID: 19621072 PMC2707599

[23] Sterne JAC Savović J Page MJ Elbers RG Blencowe NS Boutron I . RoB 2: a revised tool for assessing risk of bias in randomised trials. BMJ. (2019) 366:l4898. doi: 10.1136/bmj.l4898, PMID: 31462531

[24] Jiang B Pang J Li J Mi L Ru D Feng J . The effects of organic food on human health: a systematic review and meta-analysis of population-based studies. Nutr Rev. (2023) 82:1151–75. doi: 10.1093/nutrit/nuad124, PMID: 37930102

[25] Cochrane Handbook for Systematic Reviews of Interventions version 6.3 (2022). Cochrane. Available online at: www.training.cochrane.org/handbook. (Accessed April 5, 2025) WV.

[26] Schünemann HJ Oxman AD Brozek J Glasziou P Jaeschke R Vist GE . Grading quality of evidence and strength of recommendations for diagnostic tests and strategies. BMJ. (2008) 336:1106–10. doi: 10.1136/bmj.39500.677199.AE, PMID: 18483053 PMC2386626

[27] Argyrou C Karlafti E Lampropoulou-Adamidou K Tournis S Makris K Trovas G . Effect of calcium and vitamin D supplementation with and without collagen peptides on bone turnover in postmenopausal women with osteopenia. J Musculoskelet Neuronal Interact. (2020) 20:12–7. doi: 10.1016/j.jocd.2021.11.011, PMID: 32131366 PMC7104583

[28] Basat H Esmaeilzadeh S Eskiyurt N . The effects of strengthening and high-impact exercises on bone metabolism and quality of life in postmenopausal women: a randomized controlled trial. J Back Musculoskelet Rehabil. (2013) 26:427–35. doi: 10.3233/bmr-130402, PMID: 23948830

[29] Bolton KL Egerton T Wark J Wee E Matthews B Kelly A . Effects of exercise on bone density and falls risk factors in post-menopausal women with osteopenia: a randomised controlled trial. J Sci Med sport. (2012) 15:102–9. doi: 10.1016/j.jsams.2011.08.007, PMID: 21996058

[30] Castelo-Branco C Dávila J Alvarez L Balasch J . Comparison of the effects of calcium carbonate and ossein-hydroxyapatite complex on back and knee pain and quality of life in osteopenic perimenopausal women. Maturitas. (2015) 81:76–82. doi: 10.1016/j.maturitas.2015.02.265, PMID: 25819354

[31] Cheung AM Tile L Lee Y Tomlinson G Hawker G Scher J . Vitamin K supplementation in postmenopausal women with osteopenia (ECKO trial): a randomized controlled trial. PloS Med. (2008) 5:e196. doi: 10.1371/journal.pmed.0050196, PMID: 18922041 PMC2566998

[32] Cúneo F Costa-Paiva L Pinto-Neto AM Morais SS Amaya-Farfan J . Effect of dietary supplementation with collagen hydrolysates on bone metabolism of postmenopausal women with low mineral density. Maturitas. (2010) 65:253–7. doi: 10.1016/j.maturitas.2009.10.002, PMID: 19892499

[33] Daly RM Gianoudis J Kersh ME Bailey CA Ebeling PR Krug R . Effects of a 12-month supervised, community-based, multimodal exercise program followed by a 6-month research-to-practice transition on bone mineral density, trabecular microarchitecture, and physical function in older adults: A randomized controlled trial. J Bone Miner Res. (2020) 35:419–29. doi: 10.1002/jbmr.3865, PMID: 31498937

[34] Elam ML Johnson SA Hooshmand S Feresin RG Payton ME Gu J . A calcium-collagen chelate dietary supplement attenuates bone loss in postmenopausal women with osteopenia: a randomized controlled trial. J Med Food. (2015) 18:324–31. doi: 10.1089/jmf.2014.0100, PMID: 25314004

[35] Filip R Possemiers S Heyerick A Pinheiro I Raszewski G Davicco MJ . Twelve-month consumption of a polyphenol extract from olive (Olea europaea) in a double blind, randomized trial increases serum total osteocalcin levels and improves serum lipid profiles in postmenopausal women with osteopenia. J Nutr Health Aging. (2015) 19:77–86. doi: 10.1007/s12603-014-0480-x, PMID: 25560820 PMC12280459

[36] Granchi D Caudarella R Ripamonti C Spinnato P Bazzocchi A Massa A . Potassium citrate supplementation decreases the biochemical markers of bone loss in a group of osteopenic women: the results of a randomized, double-blind, placebo-controlled pilot study. Nutrients. (2018) 10:1293. doi: 10.3390/nu10091293, PMID: 30213095 PMC6164684

[37] Hooshmand S Kern M Metti D Shamloufard P Chai SC Johnson SA . The effect of two doses of dried plum on bone density and bone biomarkers in osteopenic postmenopausal women: a randomized, controlled trial. Osteoporos Int. (2016) 27:2271–9. doi: 10.1007/s00198-016-3524-8, PMID: 26902092

[38] Jafarnejad S Djafarian K Fazeli MR Yekaninejad MS Rostamian A Keshavarz SA . Effects of a multispecies probiotic supplement on bone health in osteopenic postmenopausal women: A randomized, double-blind, controlled trial. J Am Coll Nutr. (2017) 36:497–506. doi: 10.1080/07315724.2017.1318724, PMID: 28628374

[39] Kim KH Lee HB . Effects of circuit training interventions on bone metabolism markers and bone density of old women with osteopenia. J Exercise rehabilitation. (2019) 15:302–7. doi: 10.12965/jer.1836640.320, PMID: 31111017 PMC6509451

[40] Lambert MNT Thybo CB Lykkeboe S Rasmussen LM Frette X Christensen LP . Combined bioavailable isoflavones and probiotics improve bone status and estrogen metabolism in postmenopausal osteopenic women: a randomized controlled trial. Am J Clin Nutr. (2017) 106:909–20. doi: 10.3945/ajcn.117.153353, PMID: 28768651

[41] Lecomte M Tomassi D Rizzoli R Tenon M Berton T Harney S . Effect of a hop extract standardized in 8-prenylnaringenin on bone health and gut microbiome in postmenopausal women with osteopenia: A one-year randomized, double-blind, placebo-controlled trial. Nutrients. (2023) 15:2688. doi: 10.3390/nu15122688, PMID: 37375599 PMC10304064

[42] Li J Gu Q Li R Wang R Cai Y Huang Y . Effect of Yi Jin Jing exercise plus Elastic Band Resistance exercise on overall bone mineral density in postmenopausal women. J Sci Med Sport. (2023) 26:87–92. doi: 10.1016/j.jsams.2023.01.006, PMID: 36707306

[43] Mainini G Rotondi M Di Nola K Pezzella MT Iervolino SA Seguino E . Oral supplementation with antioxidant agents containing alpha lipoic acid: effects on postmenopausal bone mass. Clin Exp Obstet Gynecol. (2012) 39:489–93., PMID: 23444750

[44] Maria S Swanson MH Enderby LT D’Amico F Enderby B Samsonraj RM . Melatonin-micronutrients Osteopenia Treatment Study (MOTS): a translational study assessing melatonin, strontium (citrate), vitamin D3 and vitamin K2 (MK7) on bone density, bone marker turnover and health related quality of life in postmenopausal osteopenic women following a one-year double-blind RCT and on osteoblast-osteoclast co-cultures. Aging (Albany N Y). (2017) 9:256–85. doi: 10.18632/aging.101158, PMID: 28130552 PMC5310667

[45] Norton C Hettiarachchi M Cooke R Kozior M Kontro H Daniel R . Effect of 24-week, late-evening ingestion of a calcium-fortified, milk-based protein matrix on biomarkers of bone metabolism and site-specific bone mineral density in postmenopausal women with osteopenia. Nutrients. (2022) 14:3486. doi: 10.3390/nu14173486, PMID: 36079744 PMC9460355

[46] Rønn SH Harsløf T Oei L Pedersen SB Langdahl BL . The effect of vitamin MK-7 on bone mineral density and microarchitecture in postmenopausal women with osteopenia, a 3-year randomized, placebo-controlled clinical trial. Osteoporos Int. (2021) 32:185–91. doi: 10.1007/s00198-020-05638-z, PMID: 33030563

[47] Sales LP Pinto AJ Rodrigues SF Alvarenga JC Gonçalves N Sampaio-Barros MM . Creatine supplementation (3 g/d) and bone health in older women: A 2-year, randomized, placebo-controlled trial. journals gerontology Ser A Biol Sci Med Sci. (2020) 75:931–8. doi: 10.1093/gerona/glz162, PMID: 31257405

[48] Shen CL Chyu MC Yeh JK Zhang Y Pence BC Felton CK . Effect of green tea and Tai Chi on bone health in postmenopausal osteopenic women: a 6-month randomized placebo-controlled trial. Osteoporos Int. (2012) 23:1541–52. doi: 10.1007/s00198-011-1731-x, PMID: 21766228 PMC3288336

[49] Vallibhakara SA Nakpalat K Sophonsritsuk A Tantitham C Vallibhakara O . Effect of vitamin E supplement on bone turnover markers in postmenopausal osteopenic women: A double-blind, randomized, placebo-controlled trial. Nutrients. (2021) 13:4226. doi: 10.3390/nu13124226, PMID: 34959779 PMC8709036

[50] Wen HJ Huang TH Li TL Chong PN Ang BS . Effects of short-term step aerobics exercise on bone metabolism and functional fitness in postmenopausal women with low bone mass. Osteoporosis Int. (2017) 28:539–47. doi: 10.1007/s00198-016-3759-4, PMID: 27613719

[51] Lee D-H Kim M . Comparative study of lumbar bone mineral content using DXA and CT Hounsfield unit values in chest CT. BMC Musculoskelet Disord. (2023) 24:94. doi: 10.1186/s12891-023-06159-6, PMID: 36737729 PMC9898970

[52] Eslamipour F Gheitasi M Hovanloo F Yaghoubitajani Z . High versus low-intensity resistance training on bone mineral density and content acquisition by postmenopausal women with osteopenia: A randomized controlled trial. Med J Islamic Republic Iran. (2023) 37:126. doi: 10.47176/mjiri.37.126, PMID: 38318407 PMC10843212

[53] Kemmler W Lauber D Weineck J Hensen J Kalender W Engelke K . Benefits of 2 years of intense exercise on bone density, physical fitness, and blood lipids in early postmenopausal osteopenic women: results of the erlangen fitness osteoporosis prevention study (EFOPS). Arch Intern Med. (2004) 164:1084–91. doi: 10.1001/archinte.164.10.1084, PMID: 15159265

[54] Kanis JA . Assessment of fracture risk and its application to screening for postmenopausal osteoporosis: synopsis of a WHO report. WHO Study Group. Osteoporos Int. (1994) 4:368–81 doi: 10.1007/BF0162220, PMID: 7696835

[55] Shojaa M von Stengel S Kohl M Schoene D Kemmler W . Effects of dynamic resistance exercise on bone mineral density in postmenopausal women: a systematic review and meta-analysis with special emphasis on exercise parameters. Osteoporos Int. (2020) 31:1427–44. doi: 10.1007/s00198-020-05441-w, PMID: 32399891 PMC7360540

[56] Zhang L Zheng YL Wang R Wang XQ Zhang H . Exercise for osteoporosis: A literature review of pathology and mechanism. Front Immunol. (2022) 13:1005665. doi: 10.3389/fimmu.2022.1005665, PMID: 36164342 PMC9509020

[57] Pegreffi F Donati Zeppa S Gervasi M Fernández-Peña E Annibalini G Bartolacci A . A snapshot of vitamin D status, performance, blood markers, and dietary habits in runners and non-runners. Nutrients. (2024) 16:3912. doi: 10.3390/nu16223912, PMID: 39599698 PMC11597173

[58] Pinheiro MB Oliveira J Bauman A Fairhall N Kwok W Sherrington C . Evidence on physical activity and osteoporosis prevention for people aged 65+ years: a systematic review to inform the WHO guidelines on physical activity and sedentary behaviour. Int J Behav Nutr Phys Activity. (2020) 17:150. doi: 10.1186/s12966-020-01040-4, PMID: 33239014 PMC7690138

[59] Zhang S Huang X Zhao X Li B Cai Y Liang X . Effect of exercise on bone mineral density among patients with osteoporosis and osteopenia: A systematic review and network meta-analysis. J Clin Nurs. (2022) 31:2100–11. doi: 10.1111/jocn.16101, PMID: 34725872

[60] Dolan E Dumas A Keane KM Bestetti G Freitas LHM Gualano B . The bone biomarker response to an acute bout of exercise: A systematic review with meta-analysis. Sports Med. (2022) 52:2889–908. doi: 10.1007/s40279-022-01718-8, PMID: 35870108

[61] Smith C Tacey A Mesinovic J Scott D Lin X Brennan-Speranza TC . The effects of acute exercise on bone turnover markers in middle-aged and older adults: A systematic review. Bone. (2021) 143:115766. doi: 10.1016/j.bone.2020.115766, PMID: 33227507

[62] Singh S Kumar D Lal AK . Serum osteocalcin as a diagnostic biomarker for primary osteoporosis in women. J Clin Diagn Res. (2015) 9:Rc04–7. doi: 10.7860/jcdr/2015/14857.6318, PMID: 26436008 PMC4576601

[63] Martiniakova M Biro R Kovacova V Babikova M Zemanova N Mondockova V . Current knowledge of bone-derived factor osteocalcin: its role in the management and treatment of diabetes mellitus, osteoporosis, osteopetrosis and inflammatory joint diseases. J Mol Med (Berl). (2024) 102:435–52. doi: 10.1007/s00109-024-02418-8, PMID: 38363329 PMC10963459

[64] Kohrt WM Bloomfield SA Little KD Nelson ME Yingling VR . American College of Sports Medicine Position Stand: physical activity and bone health. Med Sci Sports Exerc. (2004) 36:1985–96. doi: 10.1249/01.mss.0000142662.21767.58, PMID: 15514517

[65] Daly RM Dalla Via J Duckham RL Fraser SF Helge EW . Exercise for the prevention of osteoporosis in postmenopausal women: an evidence-based guide to the optimal prescription. Braz J Phys Ther. (2019) 23:170–80. doi: 10.1016/j.bjpt.2018.11.011, PMID: 30503353 PMC6429007

[66] Angulo J El Assar M Álvarez-Bustos A Rodríguez-Mañas L . Physical activity and exercise: Strategies to manage frailty. Redox Biol. (2020) 35:101513. doi: 10.1016/j.redox.2020.101513, PMID: 32234291 PMC7284931

[67] Brooke-Wavell K Skelton DA Barker KL Clark EM De Biase S Arnold S . Strong, steady and straight: UK consensus statement on physical activity and exercise for osteoporosis. Br J Sports Med. (2022) 56:837–46. doi: 10.1136/bjsports-2021-104634, PMID: 35577538 PMC9304091

[68] Zhao R Zhang M Zhang Q . The effectiveness of combined exercise interventions for preventing postmenopausal bone loss: A systematic review and meta-analysis. J Orthop Sports Phys Ther. (2017) 47:241–51. doi: 10.2519/jospt.2017.6969, PMID: 28257620

[69] Agostini D Zeppa Donati S Lucertini F Annibalini G Gervasi M Ferri Marini C . Muscle and bone health in postmenopausal women: role of protein and vitamin D supplementation combined with exercise training. Nutrients. (2018) 10:1103. doi: 10.3390/nu10081103, PMID: 30115856 PMC6116194

[70] Vermeer C . Vitamin K: the effect on health beyond coagulation - an overview. Food Nutr Res. (2012) 56. doi: 10.3402/fnr.v56i0.5329, PMID: 22489224 PMC3321262

[71] Fusaro M Mereu MC Aghi A Iervasi G Gallieni M . Vitamin K and bone. Clin cases mineral Bone Metab. (2017) 14:200–6. doi: 10.11138/ccmbm/2017.14.1.200 PMC5726210 29263734

[72] Mott A Bradley T Wright K Cockayne ES Shearer MJ Adamson J . Effect of vitamin K on bone mineral density and fractures in adults: an updated systematic review and meta-analysis of randomised controlled trials. Osteoporos Int. (2019) 30:1543–59. doi: 10.1007/s00198-019-04949-0, PMID: 31076817

[73] Salma Ahmad SS Karim S Ibrahim IM Alkreathy HM Alsieni M . Effect of vitamin K on bone mineral density and fracture risk in adults: systematic review and meta-analysis. Biomedicines. (2022) 10:1048. doi: 10.3390/biomedicines10051048, PMID: 35625785 PMC9138595

[74] Huang ZB Wan SL Lu YJ Ning L Liu C Fan SW . Does vitamin K2 play a role in the prevention and treatment of osteoporosis for postmenopausal women: a meta-analysis of randomized controlled trials. Osteoporos Int. (2015) 26:1175–86. doi: 10.1007/s00198-014-2989-6, PMID: 25516361

[75] Fusaro M Cianciolo G Brandi ML Ferrari S Nickolas TL Tripepi G . Vitamin K and osteoporosis. Nutrients. (2020) 12:3625. doi: 10.3390/nu12123625, PMID: 33255760 PMC7760385

[76] Kim M Na W Sohn C . Vitamin K1 (phylloquinone) and K2 (menaquinone-4) supplementation improves bone formation in a high-fat diet-induced obese mice. J Clin Biochem Nutr. (2013) 53:108–13. doi: 10.3164/jcbn.13-25, PMID: 24062608 PMC3774927

[77] Kruger MC Booth CL Coad J Schollum LM Kuhn-Sherlock B Shearer MJ . Effect of calcium fortified milk supplementation with or without vitamin K on biochemical markers of bone turnover in premenopausal women. Nutrition. (2006) 22:1120–8. doi: 10.1016/j.nut.2006.08.008, PMID: 17030114

[78] Akbari S Rasouli-Ghahroudi AA . Vitamin K and bone metabolism: A review of the latest evidence in preclinical studies. BioMed Res Int. (2018) 2018:4629383. doi: 10.1155/2018/4629383, PMID: 30050932 PMC6040265

[79] Kuang X Liu C Guo X Li K Deng Q Li D . The combination effect of vitamin K and vitamin D on human bone quality: a meta-analysis of randomized controlled trials. Food Funct. (2020) 11:3280–97. doi: 10.1039/c9fo03063h, PMID: 32219282

[80] Hu L Ji J Li D Meng J Yu B . The combined effect of vitamin K and calcium on bone mineral density in humans: a meta-analysis of randomized controlled trials. J Orthop Surg Res. (2021) 16:592. doi: 10.1186/s13018-021-02728-4, PMID: 34649591 PMC8515712

[81] Nair P Orford N Kerschan-Schindl K . Micronutrient intake to protect against osteoporosis during and after critical illness. Curr Opin Clin Nutr Metab Care. (2023) 26:557–63. doi: 10.1097/mco.0000000000000979, PMID: 37650707

[82] Bonjour J-P Guéguen L Palacios C Shearer MJ Weaver CM . Minerals and vitamins in bone health: the potential value of dietary enhancement. Br J Nutr. (2009) 101:1581–96. doi: 10.1017/S0007114509311721, PMID: 19335926

[83] Townsend JR Kirby TO Sapp PA Gonzalez AM Marshall TM Esposito R . Nutrient synergy: definition, evidence, and future directions. Front Nutr. (2023) 10:1279925. doi: 10.3389/fnut.2023.1279925, PMID: 37899823 PMC10600480

[84] Porfírio E Fanaro GB . Collagen supplementation as a complementary therapy for the prevention and treatment of osteoporosis and osteoarthritis: a systematic review. Rev Bras Geriatr Gerontol. (2016) 19:153–64. doi: 10.1590/1809-9823.2016.14145

[85] Pacifici R . Estrogen deficiency, T cells and bone loss. Cell Immunol. (2008) 252:68–80. doi: 10.1016/j.cellimm.2007.06.008, PMID: 17888417

[86] Salvio G Ciarloni A Gianfelice C Lacchè F Sabatelli S Giacchetti G . The effects of polyphenols on bone metabolism in postmenopausal women: systematic review and meta-analysis of randomized control trials. Antioxidants (Basel). (2023) 12:1830. doi: 10.3390/antiox12101830, PMID: 37891909 PMC10604028

[87] Niwano Y Kohzaki H Shirato M Shishido S Nakamura K . Anti-osteoporotic mechanisms of polyphenols elucidated based on *in vivo* studies using ovariectomized animals. Antioxidants (Basel Switzerland). (2022) 11:217. doi: 10.3390/antiox11020217, PMID: 35204100 PMC8868308

[88] Hanga-Farcaș A Miere Groza F Filip GA Clichici S Fritea L Vicaș LG . Phytochemical compounds involved in the bone regeneration process and their innovative administration: A systematic review. Plants (Basel Switzerland). (2023) 12:2055. doi: 10.3390/plants12102055, PMID: 37653972 PMC10222459

[89] Hooshmand S Arjmandi BH . Viewpoint: Dried plum, an emerging functional food that may effectively improve bone health. Ageing Res Rev. (2009) 8:122–7. doi: 10.1016/j.arr.2009.01.002, PMID: 19274852

[90] van den Heuvel EGHM Steijns JMJM . Dairy products and bone health: how strong is the scientific evidence? Nutr Res Rev. (2018) 31:164–78. doi: 10.1017/S095442241800001X, PMID: 29560832

[91] Agostini D Gervasi M Ferrini F Bartolacci A Stranieri A Piccoli G . An integrated approach to skeletal muscle health in aging. Nutrients. (2023) 15:1802. doi: 10.3390/nu15081802, PMID: 37111021 PMC10141535

[92] Wang L Hu YQ Zhao ZJ Zhang HY Gao B Lu WG . Screening and validation of serum protein biomarkers for early postmenopausal osteoporosis diagnosis. Mol Med Rep. (2017) 16:8427–33. doi: 10.3892/mmr.2017.7620, PMID: 28983612

[93] Zoch ML Clemens TL Riddle RC . New insights into the biology of osteocalcin. Bone. (2016) 82:42–9. doi: 10.1016/j.bone.2015.05.046, PMID: 26055108 PMC4670816

[94] Gundberg CM Looker AC Nieman SD Calvo MS . Patterns of osteocalcin and bone specific alkaline phosphatase by age, gender, and race or ethnicity. Bone. (2002) 31:703–8. doi: 10.1016/s8756-3282(02)00902-x, PMID: 12531565

[95] Schini M Vilaca T Gossiel F Salam S Eastell R . Bone turnover markers: basic biology to clinical applications. Endocrine Rev. (2022) 44:417–73. doi: 10.1210/endrev/bnac031, PMID: 36510335 PMC10166271

[96] McCormick RK . Osteoporosis: integrating biomarkers and other diagnostic correlates into the management of bone fragility. Altern Med Rev. (2007) 12:113–45., PMID: 17604458

[97] Edwards MH Jameson K Denison H Harvey NC Sayer AA Dennison EM . Clinical risk factors, bone density and fall history in the prediction of incident fracture among men and women. Bone. (2013) 52:541–7. doi: 10.1016/j.bone.2012.11.006, PMID: 23159464 PMC3654628

[98] Bartl R Frisch B . Osteoporosis: Diagnosis, Prevention, Therapy Version 2. Heidelberg: Springer Berlin (2009). doi: 10.1007/978-3-540-79527-8

[99] Ji MX Yu Q . Primary osteoporosis in postmenopausal women. Chronic Dis Trans Med. (2015) 1:9–13. doi: 10.1016/j.cdtm.2015.02.006, PMID: 29062981 PMC5643776

[100] Feldstein A Elmer PJ Orwoll E Herson M Hillier T . Bone mineral density measurement and treatment for osteoporosis in older individuals with fractures: a gap in evidence-based practice guideline implementation. Arch Intern Med. (2003) 163:2165–72. doi: 10.1001/archinte.163.18.2165, PMID: 14557214

