# Supplementary material for: The effect of non-pharmacological interventions on bone health among patients with low bone mass: a systematic review and meta-analysis
Source: Front Endocrinol (Lausanne). 2025 Dec 10;16:1612739. doi: 10.3389/fendo.2025.1612739 (PMC12727616; doi:10.3389/fendo.2025.1612739)
Supplement: Supplementary file 1 [file Table1.docx]

Supplementary Material

# Supplementary Figures and Tables

Table S1. PRISMA check list (2020)

| **Section and Topic** | **Item #** | **Checklist item** | **Location where item is reported** |
| --- | --- | --- | --- |
| **TITLE** | | |  |
| Title | 1 | Identify the report as a systematic review. | Page 1 |
| **ABSTRACT** | | |  |
| Abstract | 2 | See the PRISMA 2020 for Abstracts checklist. | Page 3 |
| **INTRODUCTION** | | |  |
| Rationale | 3 | Describe the rationale for the review in the context of existing knowledge. | Page 4–5 |
| Objectives | 4 | Provide an explicit statement of the objective(s) or question(s) the review addresses. | Page 5–6 |
| **METHODS** | | |  |
| Eligibility criteria | 5 | Specify the inclusion and exclusion criteria for the review and how studies were grouped for the syntheses. | Page 6–7 |
| Information sources | 6 | Specify all databases, registers, websites, organisations, reference lists and other sources searched or consulted to identify studies. Specify the date when each source was last searched or consulted. | Page 6 |
| Search strategy | 7 | Present the full search strategies for all databases, registers and websites, including any filters and limits used. | Supplementary Table S2 |
| Selection process | 8 | Specify the methods used to decide whether a study met the inclusion criteria of the review, including how many reviewers screened each record and each report retrieved, whether they worked independently, and if applicable, details of automation tools used in the process. | Page 6 and Table 1 |
| Data collection process | 9 | Specify the methods used to collect data from reports, including how many reviewers collected data from each report, whether they worked independently, any processes for obtaining or confirming data from study investigators, and if applicable, details of automation tools used in the process. | Page 6 |
| Data items | 10a | List and define all outcomes for which data were sought. Specify whether all results that were compatible with each outcome domain in each study were sought (e.g. for all measures, time points, analyses), and if not, the methods used to decide which results to collect. | Page 7 |
|  | 10b | List and define all other variables for which data were sought (e.g. participant and intervention characteristics, funding sources). Describe any assumptions made about any missing or unclear information. | Page 7 |
| Study risk of bias assessment | 11 | Specify the methods used to assess risk of bias in the included studies, including details of the tool(s) used, how many reviewers assessed each study and whether they worked independently, and if applicable, details of automation tools used in the process. | Page 7–8 |
| Effect measures | 12 | Specify for each outcome the effect measure(s) (e.g. risk ratio, mean difference) used in the synthesis or presentation of results. | Page 8-9 |
| Synthesis methods | 13a | Describe the processes used to decide which studies were eligible for each synthesis (e.g. tabulating the study intervention characteristics and comparing against the planned groups for each synthesis (item #5)). | Page 8-9 |
|  | 13b | Describe any methods required to prepare the data for presentation or synthesis, such as handling of missing summary statistics, or data conversions. | Page 8-9 |
|  | 13c | Describe any methods used to tabulate or visually display results of individual studies and syntheses. | Page 8-9 |
|  | 13d | Describe any methods used to synthesize results and provide a rationale for the choice(s). If meta-analysis was performed, describe the model(s), method(s) to identify the presence and extent of statistical heterogeneity, and software package(s) used. | Page 8-9 |
|  | 13e | Describe any methods used to explore possible causes of heterogeneity among study results (e.g. subgroup analysis, meta-regression). | Page 9 |
|  | 13f | Describe any sensitivity analyses conducted to assess robustness of the synthesized results. | / |
| Reporting bias assessment | 14 | Describe any methods used to assess risk of bias due to missing results in a synthesis (arising from reporting biases). | Page 7–8 |
| Certainty assessment | 15 | Describe any methods used to assess certainty (or confidence) in the body of evidence for an outcome. | Page 9 |
| **RESULTS** | | |  |
| Study selection | 16a | Describe the results of the search and selection process, from the number of records identified in the search to the number of studies included in the review, ideally using a flow diagram. | Page 10 and Figure 1 |
|  | 16b | Cite studies that might appear to meet the inclusion criteria, but which were excluded, and explain why they were excluded. | Page 10 and Figure 1 |
| Study characteristics | 17 | Cite each included study and present its characteristics. | Page 10 and Supplementary Table 2 |
| Risk of bias in studies | 18 | Present assessments of risk of bias for each included study. | Figure 2 |
| Results of individual studies | 19 | For all outcomes, present, for each study: (a) summary statistics for each group (where appropriate) and (b) an effect estimate and its precision (e.g. confidence/credible interval), ideally using structured tables or plots. | Table 2, Table 3, Figure 3 |
| Results of syntheses | 20a | For each synthesis, briefly summarise the characteristics and risk of bias among contributing studies. | Table 2, Figure 2 |
|  | 20b | Present results of all statistical syntheses conducted. If meta-analysis was done, present for each the summary estimate and its precision (e.g. confidence/credible interval) and measures of statistical heterogeneity. If comparing groups, describe the direction of the effect. | Figure 3 |
|  | 20c | Present results of all investigations of possible causes of heterogeneity among study results. | Table 2, |
|  | 20d | Present results of all sensitivity analyses conducted to assess the robustness of the synthesized results. | / |
| Reporting biases | 21 | Present assessments of risk of bias due to missing results (arising from reporting biases) for each synthesis assessed. | Page 24–25 |
| Certainty of evidence | 22 | Present assessments of certainty (or confidence) in the body of evidence for each outcome assessed. | Page 24–25 |
| **DISCUSSION** | | |  |
| Discussion | 23a | Provide a general interpretation of the results in the context of other evidence. | Page 35–45 |
|  | 23b | Discuss any limitations of the evidence included in the review. | Page 45–46 |
|  | 23c | Discuss any limitations of the review processes used. | Page 45–46 |
|  | 23d | Discuss implications of the results for practice, policy, and future research. | Page 35–45 |
| **OTHER INFORMATION** | | |  |
| Registration and protocol | 24a | Provide registration information for the review, including register name and registration number, or state that the review was not registered. | Page 6 |
|  | 24b | Indicate where the review protocol can be accessed, or state that a protocol was not prepared. | Page 6 |
|  | 24c | Describe and explain any amendments to information provided at registration or in the protocol. | Page 6 |
| Support | 25 | Describe sources of financial or non-financial support for the review, and the role of the funders or sponsors in the review. | Page 48 |
| Competing interests | 26 | Declare any competing interests of review authors. | Page 48 |
| Availability of data, code and other materials | 27 | Report which of the following are publicly available and where they can be found: template data collection forms; data extracted from included studies; data used for all analyses; analytic code; any other materials used in the review. | Page 7–8 |

*From:*  Page MJ, McKenzie JE, Bossuyt PM, Boutron I, Hoffmann TC, Mulrow CD, et al. The PRISMA 2020 statement: an updated guideline for reporting systematic reviews. BMJ 2021;372:n71. doi: 10.1136/bmj.n71

For more information, visit: <http://www.prisma-statement.org/>

Table S2. Search strategy

| **Database** | **Search strategy** |
| --- | --- |
| **PubMed** | ((borderline[tiab] OR critical[tiab] OR subclinical[tiab] OR “pre-”[tiab] OR prodromal[tiab] OR early[tiab] OR “early-stage”[tiab]) AND (“osteoporosis”[tiab] OR "Osteoporosis"[Mesh] OR “rarefaction of bone”[tiab]) OR “bone fragility”[tiab] OR “bone loss”[tiab] OR “decreased bone”[tiab] OR “decreased bone strength”[tiab] OR “osteopenia”[tiab] OR “bone microstructure degeneration”[tiab] OR “decreased bone mineral”[tiab] OR “decreased BMC”[tiab] OR “decreased BMD”[tiab] OR “decreased bone density”[tiab] OR “low bone mass” [tiab] OR “bone microstructure degeneration”[tiab] OR “destruction of bone microstructure”[tiab] OR “degeneration of bone microstructure”[tiab] OR “bone tissue microstructure degradation”[tiab]))  AND (lifestyle*[tiab] OR lifestyle[Mesh] OR “life style*”[tiab] OR “physical activit*”[tiab] OR “physical activity”[Mesh] OR exercise*[tiab] OR exercise[Mesh] OR sport*[tiab] OR sport[Mesh] OR “sedentary lifestyle”[Mesh] OR “sedentary time”[Mesh] OR “physical inactivit*”[tiab] OR sleep[tiab] OR sleep[Mesh] OR sleeping[tiab] OR nap*[tiab] OR smoke[tiab] OR smoking[tiab] OR smoking[Mesh] OR “cigarette smoking”[Mesh] OR cigarette[tiab] OR tobacco[tiab] OR “tobacco use”[Mesh] OR drink[tiab] OR drinking[tiab] OR “drinking behavior”[Mesh] OR alcohol[tiab] OR alcohol[Mesh] OR diet*[tiab] OR dietary[tiab] OR nutrient*[tiab] OR nutrition[tiab] OR nutrition[Mesh] OR food*[tiab] OR energy[tiab] OR energy[Mesh]) OR intake*[tiab] OR consumption*[tiab] OR supplement*[tiab] OR “dietary supplement”[Mesh] OR supplementation*[tiab])  AND (intervention*[tiab] OR intervention[Mesh] OR “controlled clinical trial”[tiab] OR “randomized controlled trial”[tiab] OR “clinical trial”[tiab] OR random*[tiab] OR trial*[tiab])  AND (adults[tiab] OR “18 years and above”[tiab] OR “above 18 years”[tiab] OR “over 18 years”[tiab] OR men[tiab] OR women[tiab] OR “middle age*”[tiab] OR “middle-aged”[tiab] OR “middle-aged”[Mesh] OR mid-aged[tiab] OR “old people”[tiab] OR “the aged”[tiab] OR “aged people”[tiab] OR “senior people”[tiab] OR “senior citizen*”[tiab] OR elder*[tiab] OR “the elderly”[tiab]) |
| **Web of science** | ((TI= (borderline OR critical OR subclinical OR “pre-” OR prodromal OR early OR “early-stage”) OR AB= (borderline OR critical OR subclinical OR “pre-” OR prodromal OR early OR “early-stage”)) AND (TI=(“osteoporosis” OR “rarefaction of bone”) OR AB= (“osteoporosis” OR “rarefaction of bone”))) OR (TI=(“bone fragility” OR “bone loss” OR “decreased bone” OR “decreased bone strength” OR “osteopenia” OR “bone microstructure degeneration” OR “decreased bone mineral” OR “decreased BMC” OR “decreased BMD” OR “decreased bone density” OR “low bone mass” OR “bone microstructure degeneration” OR “destruction of bone microstructure” OR “degeneration of bone microstructure” OR “bone tissue microstructure degradation”) OR AB= (“bone fragility” OR “bone loss” OR “decreased bone” OR “decreased bone strength” OR “osteopenia” OR “bone microstructure degeneration” OR “decreased bone mineral” OR “decreased BMC” OR “decreased BMD” OR “decreased bone density” OR “low bone mass” OR “bone microstructure degeneration” OR “destruction of bone microstructure” OR “degeneration of bone microstructure” OR “bone tissue microstructure degradation”))  AND (TI= (lifestyle* OR “life style*” OR “physical activit*” OR exercise* OR sport* OR sit OR sitting OR sedentariness OR sedentary OR “physical inactivit*” OR sleep OR sleeping OR nap* OR smoke OR smoking OR cigarette OR tobacco OR drink OR drinking OR alcohol OR diet* OR dietary OR nutrient* OR nutrition OR food* OR eat OR eating OR energy OR intake* OR consumption* OR supplement* OR supplementation*) OR AB= (lifestyle* OR “life style*” OR “physical activit*” OR exercise* OR sport* OR sit OR sitting OR sedentariness OR sedentary OR “physical inactivit*” OR sleep OR sleeping OR nap* OR smoke OR smoking OR cigarette OR tobacco OR drink OR drinking OR alcohol OR diet* OR dietary OR nutrient* OR nutrition OR food* OR eat OR eating OR energy OR intake* OR consumption* OR supplement* OR supplementation*))  AND (TI=(intervention* OR “controlled clinical trial” OR “randomized controlled trial” OR “clinical trial” OR “random*” OR “trial*”) OR AB=(intervention* OR “controlled clinical trial” OR “randomized controlled trial” OR “clinical trial” OR “random*” OR “trial*”))  AND (TI=(adults OR “18 years and above” OR “above 18 years” OR “over 18 years” OR men OR women OR “middle age*” OR “middle-aged” OR mid-aged OR “old people” OR “the aged” OR “aged people” OR “senior people” OR “senior citizen*” OR elder* OR “the elderly”) OR AB=(adults OR “18 years and above” OR “above 18 years” OR “over 18 years” OR men OR women OR “middle age*” OR “middle-aged” OR mid-aged OR “old people” OR “the aged” OR “aged people” OR “senior people” OR “senior citizen*” OR elder* OR “the elderly”)) |
| **Embase** | (((borderline:ab,ti,kw OR critical:ab,ti,kw OR subclinical:ab,ti,kw OR ‘pre-‘:ab,ti,kw OR prodromal:ab,ti,kw OR early:ab,ti,kw OR ‘early-stage’:ab,ti,kw) AND (‘osteoporosis’:ab,ti,kw OR ‘osteoporosis’/exp OR ‘rarefaction of bone’:ab,ti,kw)) OR (‘bone fragility’:ab,ti,kw OR ‘bone loss’:ab,ti,kw OR ‘decreased bone’:ab,ti,kw OR ‘decreased bone strength’:ab,ti,kw OR ‘osteopenia’:ab,ti,kw OR ‘osteopenia’/exp OR ‘bone microstructure degeneration’:ab,ti,kw OR ‘decreased bone mineral’:ab,ti,kw OR ‘decreased BMC’:ab,ti,kw OR ‘decreased BMD’:ab,ti,kw OR ‘decreased bone density’:ab,ti,kw OR ‘low bone mass’:ab,ti,kw OR ‘bone microstructure degeneration’:ab,ti,kw OR ‘destruction of bone microstructure’:ab,ti,kw OR ‘degeneration of bone microstructure’:ab,ti,kw OR ‘bone tissue microstructure degradation’:ab,ti,kw))  AND (lifestyle*:ab,ti,kw OR lifestyle/exp OR ‘life style*’:ab,ti,kw OR ‘physical activit*’:ab,ti,kw OR ‘physical activity’/exp OR exercise*:ab,ti,kw OR exercise/exp OR sport*:ab,ti,kw OR sport/exp OR ‘sedentary lifestyle’/exp OR ‘sedentary time’/exp OR ‘physical inactivit*’:ab,ti,kw OR sleep:ab,ti,kw OR sleep/exp OR sleeping:ab,ti,kw OR nap*:ab,ti,kw OR smoke:ab,ti,kw OR smoking:ab,ti,kw OR smoking/exp OR ‘cigarette smoking’/exp OR cigarette:ab,ti,kw OR tobacco:ab,ti,kw OR ‘tobacco use’/exp OR drink:ab,ti,kw OR drinking:ab,ti,kw OR ‘drinking behavior’/exp OR alcohol:ab,ti,kw OR alcohol/exp OR diet*:ab,ti,kw OR dietary:ab,ti,kw OR nutrient*:ab,ti,kw OR nutrition:ab,ti,kw OR nutrition/exp OR food*:ab,ti,kw OR energy:ab,ti,kw OR energy/exp OR intake*:ab,ti,kw OR consumption*:ab,ti,kw OR supplement*:ab,ti,kw OR ‘dietary supplement’/exp OR supplementation*:ab,ti,kw)  AND (intervention*:ab,ti,kw OR intervention/exp OR ‘controlled clinical trial’:ab,ti,kw OR ‘randomized controlled trial’:ab,ti,kw OR ‘clinical trial’:ab,ti,kw OR random*:ab,ti,kw OR trial*:ab,ti,kw) AND (adults:ab,ti,kw OR “18 years and above”:ab,ti,kw OR “above 18 years”:ab,ti,kw OR “over 18 years”:ab,ti,kw OR men:ab,ti,kw OR women:ab,ti,kw OR “middle age*”:ab,ti,kw OR “middle-aged”:ab,ti,kw OR “middle-aged”/exp OR mid-aged:ab,ti,kw OR “old people”:ab,ti,kw OR “the aged”:ab,ti,kw OR “aged people”:ab,ti,kw OR “senior people”:ab,ti,kw OR “senior citizen*”:ab,ti,kw OR elder*:ab,ti,kw OR “the elderly”:ab,ti,kw)  AND (adults:ab,ti,kw OR “18 years and above”:ab,ti,kw OR “above 18 years”:ab,ti,kw OR “over 18 years”:ab,ti,kw OR men:ab,ti,kw OR women:ab,ti,kw OR “middle age*”:ab,ti,kw OR “middle-aged”:ab,ti,kw OR “middle-aged”/exp OR mid-aged:ab,ti,kw OR “old people”:ab,ti,kw OR “the aged”:ab,ti,kw OR “aged people”:ab,ti,kw OR “senior people”:ab,ti,kw OR “senior citizen*”:ab,ti,kw OR elder*:ab,ti,kw OR “the elderly”:ab,ti,kw) |
| **CINAHL** | (((TI borderline OR TI critical OR TI subclinical OR TI “pre-” OR TI prodromal OR TI early OR TI “early-stage” OR AB borderline OR AB critical OR AB subclinical OR AB “pre-” OR AB prodromal OR AB early OR AB “early-stage”) AND (TI “osteoporosis” OR MH "Osteoporosis" OR AB “osteoporosis” OR AB “rarefaction of bone”)) OR (TI “rarefaction of bone”)OR TI “bone fragility” OR TI “bone loss” OR TI “decreased bone” OR TI “decreased bone strength” OR TI “osteopenia” OR TI “bone microstructure degeneration” OR TI “decreased bone mineral” OR TI “decreased BMC” OR TI “decreased BMD” OR TI “decreased bone density” OR TI “low bone mass” OR TI “bone microstructure degeneration” OR TI “destruction of bone microstructure” OR TI “degeneration of bone microstructure” OR TI “bone tissue microstructure degradation” OR AB “bone fragility” OR AB “bone loss” OR AB “decreased bone” OR AB “decreased bone strength” OR AB “osteopenia” OR AB “bone microstructure degeneration” OR AB “decreased bone mineral” OR AB “decreased BMC” OR AB “decreased BMD” OR AB “decreased bone density” OR AB “low bone mass” OR AB “bone microstructure degeneration” OR AB “destruction of bone microstructure” OR AB “degeneration of bone microstructure” OR AB “bone tissue microstructure degradation”))  AND (TI lifestyle* OR TI “life style*” OR MH "Life Style" OR TI “physical activit*” OR TI exercise* OR MH "Exercise" OR sport* OR TI Training* OR TI sit OR TI sitting OR TI sedentariness OR TI sedentary OR MH "Sedentary Behavior" OR TI “physical inactivit*” OR TI sleep OR MH "Sleep" OR TI sleeping OR TI nap OR TI naps OR TI smoke OR TI smoking OR MH "Smoking" OR TI cigarette OR MH "Cigarette Smoking" OR TI tobacco OR MH "Tobacco Use" OR TI drink OR TI drinking OR MH "Binge Drinking" OR MH "Drinking Behavior" OR TI alcohol OR MH "Alcohol Drinking" OR TI wine OR MH "Wine" OR diet OR TI diets OR MH “diet” OR TI eat OR TI eating OR MH “eating” OR TI dietary OR TI nutrient* OR MH nutrients OR TI nutrition OR TI food* OR MH food OR TI energy OR TI intake* OR TI consumption* OR TI supplement* OR MH “dietary supplements” OR TI supplementation* OR AB lifestyle* OR AB “life style*” OR AB “physical activit*” OR AB exercise* OR sport* OR AB Training* OR AB sit OR AB sitting OR AB sedentariness OR AB sedentary OR AB “physical inactivit*” OR AB sleep OR AB sleeping OR AB nap OR AB naps OR AB smoke OR AB smoking OR AB cigarette OR AB tobacco OR AB drink OR AB drinking OR AB alcohol OR AB wine OR diet OR AB diets OR AB eat OR AB eating OR AB dietary OR AB nutrient* OR AB nutrition OR AB food* OR AB energy OR AB intake* OR AB consumption* OR AB supplement* OR AB supplementation*))  AND (TI intervention* OR TI "controlled clinical trial" OR TI "randomized controlled trial" OR TI "clinical trial" OR TI random* OR TI trial* OR AB intervention* OR AB "controlled clinical trial" OR AB "randomized controlled trial" OR AB "clinical trial" OR AB random* OR AB trial*)  AND (TI adults OR MH “Adult” OR TI “18 years and above” OR TI “above 18 years” OR TI “over 18 years” OR TI men OR MH "Men" OR TI women OR MH "Women" OR TI “middle age*” OR MH "Middle Aged" OR TI “middle-aged” OR TI mid-aged OR TI “old people” OR TI “the aged” OR MH "Aged" OR TI “aged people” OR TI “senior people” OR TI “senior citizen*” OR TI elder* OR TI “the elderly”OR AB adults OR AB “18 years and above” OR AB “above 18 years” OR AB “over 18 years” OR AB men OR AB women OR AB “middle age*” OR AB “middle-aged” OR AB mid-aged OR AB “old people” OR AB “the aged” OR AB “aged people” OR AB “senior people” OR AB “senior citizen*” OR AB elder* OR AB “the elderly”) |
